# Cumulative blood pressure load as a predictor of arterial stiffness progression and incident diabetic kidney disease: a multicenter longitudinal study

**DOI:** 10.1186/s12933-025-02785-7

**Published:** 2025-05-24

**Authors:** Cong Liu, Bangqun Ji, Yao Liang, Juan Shi, Yufan Wang, Tingyu Ke, Li Li, Dong Zhao, Yuancheng Dai, Qijuan Dong, Fengmei Xu, Ying Peng, Weiqing Wang, Qidong Zheng, Yifei Zhang

**Affiliations:** 1 Department of Endocrine and Metabolic Diseases, Shanghai Institute of Endocrine and Metabolic Diseases, Ruijin Hospital, Shanghai Jiao Tong University School of Medicine, 197 Ruijin 2nd Road, Shanghai, China; 2 Shanghai National Clinical Research Center for Metabolic Diseases, Key Laboratory for Endocrine and Metabolic Diseases of the National Health Commission of the PR China, Shanghai Key Laboratory for Endocrine Tumor, State Key Laboratory of Medical Genomics, Ruijin Hospital, Shanghai Jiao Tong University School of Medicine, 197 Ruijin 2nd Road, Shanghai, China; 3 Department of Endocrinology, Xingyi People’s Hospital, Xingyi, China; 4 Department of Internal Medicine, The Second People’s Hospital of Yuhuan, Yuhuan, China; 5 Department of Endocrinology and Metabolism, Shanghai General Hospital, Shanghai Jiao Tong University School of Medicine, Shanghai, China; 6 Department of Endocrinology, The Second Affiliated Hospital of Kunming Medical University, Kunming, China; 7 Department of Endocrinology, The First Affiliated Hospital of Ningbo University, Ningbo, China; 8 Center for Endocrine Metabolism and Immune Diseases, Beijing Luhe Hospital, Capital Medical University, Beijing, China; 9 Department of Internal Medicine of Traditional Chinese Medicine, Sheyang Diabetes Hospital, Yancheng, China; 10 Department of Endocrinology and Metabolism, People’s Hospital of Zhengzhou Affiliated, Henan University of Chinese Medicine, Zhengzhou, China; 11 Department of Endocrinology and Metabolism, Hebi Coal (Group). LTD. General Hospital, Hebi, China

**Keywords:** cumulative blood pressure load, arterial stiffness, Diabetic kidney disease, Modifiable factors, Type 2 diabetes

## Abstract

**Background:**

Elevated blood pressure (BP) is a key contributor to the progression of arterial stiffness and the incidence of diabetic kidney disease (DKD). Cumulative BP load enables the evaluation of long-term BP exposure, but the BP targets used to calculate it vary across studies. This study aimed to compare the predictive performance of cumulative BP load calculated using different clinically recommended BP targets.

**Methods:**

This multicenter longitudinal study included participants with type 2 diabetes from 10 diabetes centers. Cumulative BP load was calculated using various clinically recommended BP targets (SBP < 140 mmHg, < 130 mmHg, and < 120 mmHg). The primary outcomes were the progression of arterial stiffness, assessed by brachial-ankle pulse wave velocity (ba-PWV) changes, and the incidence of DKD, defined as an estimated glomerular filtration rate < 60 mL/min/1.73m^2^ or urine albumin-to-creatinine ratio ≥ 3.39 mg/mmol. The predictive ability of cumulative BP load calculated under different clinically recommended targets was compared using adjusted R squared (adjusted R^2^) for continuous outcomes and net reclassification improvement (NRI) for binary outcomes.

**Results:**

Among the 18,168 participants included (mean age 54.5 years; 57.5% male), 13,388 met all eligibility criteria for the analysis of arterial stiffness progression, and 11,145 for the analysis of DKD incidence. Over a mean follow-up of 3.5 years, the median ba-PWV increase was 0.19 m/s per year and 2,855 (25.6%) developed DKD. When cumulative BP load was added to a model containing traditional risk factors, the adjusted R^2^ values for predicting the absolute annual change in ba-PWV were 0.193 (95% CI 0.180–0.200), 0.184 (0.169–0.191), and 0.172 (0.158–0.180) with BP targets of SBP < 120, < 130, and < 140 mmHg, respectively. For the incidence of DKD, the NRIs were 15.8% (11.5–20.0%), 12.5% (8.3–16.7%), and 6.4% (2.3–10.6%) with BP targets of SBP < 120, < 130, and < 140 mmHg, respectively.

**Conclusion:**

Cumulative BP load is an effective indicator for predicting the progression of arterial stiffness and incidence of DKD, with the best predictive performance observed when the target SBP is set at < 120 mmHg.

**Graphical abstract:**

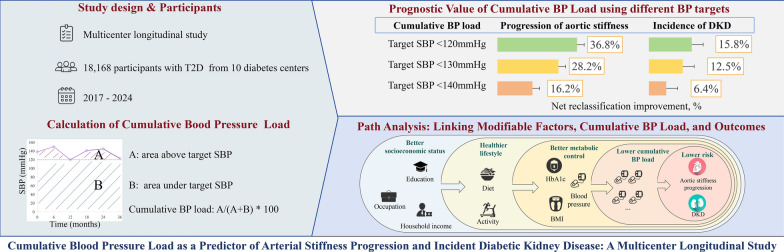

**Supplementary Information:**

The online version contains supplementary material available at 10.1186/s12933-025-02785-7.

## Research in context

**What is currently known about this topic?**

- Hypertension is a major risk factor for cardiovascular and kidney diseases.
- Single-point BP measurements poorly reflect long-term BP status.
- Cumulative BP load calculation lacks standardized target thresholds.

**What is the key research question?**

Does cumulative BP load based on different targets predict arterial stiffness progression and DKD?

**What is new?**

- Cumulative BP load based on SBP < 120 mmHg showed superior predictive performance.
- Favorable socioeconomic status was associated with lower cumulative SBP load.

**How might this study influence clinical practice?**

Cumulative BP load enhances risk prediction for arterial stiffness progression and DKD.

## Background

Diabetes has emerged as a major global public health challenge, with the number of patients expected to exceed 1.3 billion by 2050 [1]. Cardiovascular disease (CVD) remains the primary cause of morbidity and mortality among diabetic patients. Arterial stiffness, a key early indicator of vascular wall structural and functional abnormalities, is an independent predictor of cardiovascular diseases and mortality [2]. In addition, diabetic kidney disease (DKD) is a recognized risk factor for cardiovascular events [3]. Studies have demonstrated that patients with DKD have a twofold higher risk of cardiovascular mortality compared to those without DKD [4].

Hypertension is a key modifiable risk factor contributing to the development of diabetic complications [5]. Recently, a clinical trial demonstrated that intensive blood pressure (BP) management, targeting a systolic blood pressure (SBP) of less than 120 mmHg, can reduce the risk of major cardiovascular events by 21% compared to standard SBP targets (SBP < 140 mmHg) [6, 7]. However, maintaining blood pressure within recommended targets remains challenging in clinical practice, particularly for individuals with type 2 diabetes [8, 9]. Although office BP measurement is widely used in clinical practice, values obtained at a single visit may not reliably represent an individual's long-term BP exposure [10]. To overcome this limitation, recent studies have utilized various indices derived from BP measurements across multiple visits to better characterize the long-term burden of elevated BP. These indices include cumulative BP load, time at target (TITRE) of BP, and variability of BP [11–13]. Cumulative BP load is defined as the proportion of the area under the BP-time curve that lies above the clinically recommended BP target, relative to the total area under the BP-time curve [14–18]. A previous study has shown that cumulative BP load improves the prediction of cardiovascular events [16]. However, variations in recommended BP targets across different clinical guidelines and trials have led to inconsistent calculations for cumulative BP load, which may hinder its comparability and clinical application [14, 15, 19–22].

Furthermore, previous studies have shown that socioeconomic status, lifestyle, and metabolic factors are strongly associated with diabetic cardio-renal complications [23–27]. However, the role of cumulative blood pressure exposure in this relationship remains poorly understood. While research on cumulative BP load has focused on its direct associations with outcomes, it has not yet explored the upstream modifiable factors linked to this exposure. Structural equation modeling is a powerful tool to simultaneously analyze the complex relationships among multiple variables and quantify the potential pathways connecting these modifiable factors to outcomes [28, 29].

This study has two primary objectives in light of the current research landscape. First, we aim to compare the predictive value of cumulative BP load calculated using different clinically recommended BP control targets for predicting the progression of arterial stiffness and the incidence of DKD. Second, structural equation modeling will be applied to identify possible interrelationships among socioeconomic factors, lifestyle behaviors, metabolic profiles, cumulative BP load, and the risk of diabetic cardio-renal complications.

## Methods

### Study population

This study was based on a prospective, real-world cohort drawn from the National Metabolic Management Center (MMC) program, a national initiative launched in 2016 to standardize and enhance the management of diabetes and related metabolic disorders across China [30]. The program employs a unified operational model (“One Center, One Stop, One Standard Model”) to ensure consistency in diagnosis, treatment, data collection, and follow-up [30].

Participants in this analysis were recruited from 10 MMC sites located in various regions of China, including northern (Beijing), eastern (Shanghai, Zhejiang, Jiangsu), central (Henan), and southwestern (Guizhou, Yunnan) provinces. The selection of these centers has been described in the previous publication [23]. Between June 2017 and July 2024, participants aged ≥ 18 years with type 2 diabetes were enrolled. For the analysis of arterial stiffness progression, participants were included if they had at least two measurements of brachial-ankle pulse wave velocity (ba-PWV) and blood pressure records from at least four clinic visits before the final ba-PWV assessment. For the diabetic kidney disease (DKD) analysis, participants without baseline DKD—defined as estimated glomerular filtration rate (eGFR) ≥ 60 mL/min/1.73 m^2^ and urine albumin-to-creatinine ratio (UACR) < 3.39 mg/mmol—were included. These participants were also required to have at least four BP measurements before DKD onset or the final assessments of eGFR and UACR. Participants were excluded if they had a follow-up period shorter than 18 months, missing data on baseline or outcome variables (ba-PWV, eGFR, or UACR), or fewer than four BP measurements (Figure S1).

Ethical approval was granted by the Ethical Review Committee of Ruijin Hospital, affiliated with the Shanghai Jiao Tong University School of Medicine, and by the ethics committees of all other participating centers. All participants provided written informed consent before enrollment. The study design followed the Strengthening the Reporting of Observational Studies in Epidemiology (STROBE) guidelines.

### Data collection

**Fig. 1:**
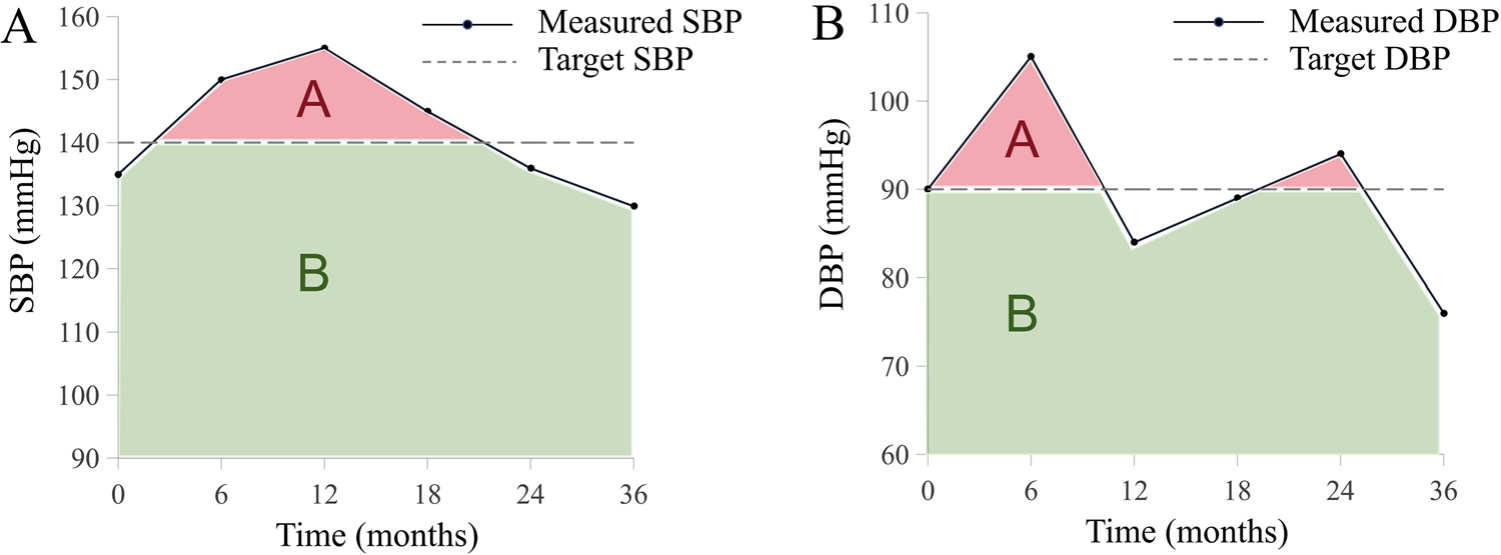
Conceptual illustration of cumulative SBP load (**A**) and cumulative DBP load (**B**) calculation. In accordance with current clinical practice guidelines, cumulative BP load was calculated based on three SBP control targets (< 120, < 130, and < 140 mmHg) and two DBP control targets (< 80 and < 90 mmHg). Dashed lines represent the clinically recommended BP targets. The red-shaded areas labeled “A” represent the area above the target BP line, while the green-shaded areas labeled “B” represent values within the target range. Cumulative BP load is defined as the proportion of area A to the total area under the BP-time curve (A + B). *Note* To enhance visual clarity, the y-axis is truncated. The calculation of cumulative BP load was performed using the entire area under the curve

At baseline, trained personnel administered comprehensive questionnaires to participants following standardized protocols. The study protocol is publicly accessible on ClinicalTrials.gov (NCT03811470). The questionnaire covered demographics, socioeconomic status, medical history, lifestyle factors, and other relevant information [30, 31]. Blood pressure was measured using two electronic BP monitors (OMRON HBP-1100 U and HBP-9031C) after participants remained seated for at least 5 min. For OMRON HBP-1100 U, an appropriate cuff size was selected based on the participant’s arm circumference. The applicable arm circumference range of OMRON HBP-9031C was 17–42 cm. All measurements were taken with the mid-arm supported at heart level. Raw measurement data were automatically uploaded to the proprietary electronic medical record system upon completion. All healthcare providers received standardized training before study initiation at each MMC site to ensure protocol adherence and measurement consistency. The cumulative blood pressure load was calculated for each participant based on all available BP measurements during follow-up. Specifically, it was defined as the percentage of the area under the BP-time curve where BP values exceeded a recommended BP control target, relative to the total area under the curve [14–18]. For SBP, cumulative SBP load was assessed using three commonly recommended control targets: SBP < 140 mmHg, < 130 mmHg, and < 120 mmHg. For DBP, cumulative DBP load was calculated using two control targets: DBP < 90 mmHg and < 80 mmHg (Fig. 1).

### Outcomes

The primary outcomes were the progression of arterial stiffness and the incidence of DKD. Arterial stiffness was assessed by measuring ba-PWV using an automatic device (BP-203RPE III, Form PWV/ABI, Omron Healthcare Co.) after participants rested supine for at least 5 min. Appropriate cuffs were placed on both upper arms and ankles to obtain brachial and tibial arterial waveforms. The ba-PWV was calculated by dividing the transmission distance between the brachium and ankle by the transmission time. The mean value of bilateral ba-PWV measurements was used for analysis [32, 33]. The incidence of DKD was defined as the presence of albuminuria (UACR ≥ 3.39 mg/mmol) or reduced eGFR (< 60 mL/min/1.73m^2^) [34].

As part of the MMC program protocol, ba-PWV was scheduled to be measured at enrollment, year 2 or year 3 of follow-up, and again in year 5 (a minimum of three measurements during the five-year timeframe if possible). DKD assessment was recommended approximately every 6 months during follow-up.

Arterial stiffness progression was evaluated using two complementary approaches. First, annual changes in ba-PWV were calculated in two forms: (1) absolute change, defined as (last ba-PWV—first ba-PWV) / time interval (in m/s per year); and (2) relative change, defined as [(last ba-PWV- first ba-PWV) / first ba-PWV] × 100/time interval (in % per year) [35, 36]. Second, participants were stratified into two subgroups based on whether their final ba-PWV measurements showed increased/consistently high or decreased/consistently low compared to their initial measurements (Figure S2). Initial and final ba-PWV measurements were divided into quartiles to establish this classification. Participants were categorized into either the increased/consistently high group (third and fourth quartiles) or the decreased/consistently low group (first and second quartiles) [35–37].

### Statistical analysis

Continuous variables were presented as mean ± SD or median (interquartile range), while categorical variables were summarized as counts and percentages. Group differences were assessed using Student's t-test, Wilcoxon rank-sum test, or chi-square test, as appropriate. Missing baseline covariate data were estimated using multiple imputations by chained equations (MICE). All covariates had missing data proportions below 10% (Table S1). Results from five imputed datasets were combined using Rubin's rules to obtain overall estimates [38].

Since cumulative BP load was calculated based on repeated BP measurements obtained at multiple clinical visits throughout the follow-up period, multivariable linear regression models were fitted using both absolute and relative annual ba-PWV change as continuous outcomes and logistic regression models were employed to evaluate the independent effects on the categorical outcomes. These analyses adjusted for baseline covariates including age, sex, diabetes duration, blood pressure, glycated hemoglobin (HbA1c), body mass index (BMI), triglycerides, total cholesterol, high-density lipoprotein cholesterol, low-density lipoprotein cholesterol, history of cardiovascular disease, history of hypertension, and use of antihypertensive medications. In addition, the number of BP measurements was included as a covariate in all models to account for differences in measurement frequency. Analyses for arterial stiffness progression were additionally adjusted for baseline ba-PWV, while models for incident DKD were adjusted for baseline eGFR. Collinearity diagnosis was examined using the variance inflation factor (VIF) and a VIF ≥ 2.5 was considered indicative of collinearity [39]. Nonlinear relationships between cumulative BP load and outcomes were assessed using restricted cubic splines (RCS), with four knots at the 5th, 35th, 65th, and 95th percentiles of cumulative BP load. The nonlinearity assumption was tested using the Wald test.

To compare the predictive performance of the cumulative BP load for the progression of arterial stiffness and the incidence of DKD, we used adjusted R squared (adjusted R^2^) and its 95% confidence interval (CI) for continuous outcomes and C-statistics, net reclassification improvement (NRI), and Akaike information criterion (AIC) for categorical outcomes [40–42].

Structural equation modeling was performed for path analysis to systematically explore the relationships among socioeconomic status, lifestyle factors, metabolic indicators, and their possible direct and indirect relationships with cumulative BP load and diabetic cardio-renal complications. Risk factors for the structural equation model were selected based on existing literature and practical considerations, including biological plausibility, statistical association with diabetic complications, modifiability, availability in the database, and low levels of missing data. A detailed summary of the selection criteria is provided in Table S2. Detailed measurements and definitions for all these modifiable factors are provided in the Supplementary Methods.

The temporal sequence determined exposure and mediating factors: education level typically preceded occupational status, household income, and lifestyle factors, which precede baseline metabolic indicators. Cumulative BP load was calculated during the follow-up period, while the progression of arterial stiffness and the incidence of DKD outcomes were determined after the final BP measurement. All continuous variables were standardized to enable a more straightforward comparison of path coefficients. Bootstrap methods were used for parameter estimation and robustness analysis, with standard errors obtained through 5,000 resampling iterations. The final model was adjusted for age and sex to reduce confounding bias and optimized based on modification indices [43]. The model fit was evaluated using several indices, including the root mean square error of approximation (RMSEA), where values of ≤ 0.06 indicate a good fit and ≤ 0.08 suggest an adequate fit; the comparative fit index (CFI), with ≥ 0.95 indicating a good fit and ≥ 0.90 representing an adequate fit; and the Tucker-Lewis index (TLI), where a value of ≥ 0.95 is considered a good fit and ≥ 0.90 is considered an adequate [44]. Path coefficients were interpreted based on their direction, magnitude, and statistical significance (*P* < 0.05), with standardized coefficients clarifying the relative impact of different predictive factors.

All statistical analyses were performed using R version 4.4.2 (R Foundation). Statistical significance was determined at a two-sided *P*-value of < 0.05.

### Sensitivity analyses

First, although we adjusted for baseline cardiovascular disease status in the primary analyses, we excluded patients with a history of cardiovascular disease from the arterial stiffness progression cohort to assess the impact of excluding this subgroup from the primary sample. Second, we stratified patients according to their baseline hypertension history and compared the optimal target values for calculating cumulative BP load between the two groups. Third, we evaluated the predictive value of two indicators, BP TITRE and BP variability, on cardio-renal outcomes. BP TITRE was defined as the percentage of days during follow-up when blood pressure remained below the BP control target [12]. Using the same methodology as for cumulative BP load, we assessed SBP TITRE with three control targets: SBP < 140 mmHg, SBP < 130 mmHg, and SBP < 120 mmHg, and DBP TITRE with two control targets: DBP < 90 mmHg and DBP < 80 mmHg. BP variability was evaluated using the standard deviation (SD) of all BP measurements to assess overall variability and average real variability (ARV) to quantify the mean absolute differences between these measurements [45]. Fourth, we conducted an additional evaluation in a subsample of participants with BP measurements recorded at baseline, 6 (± 1 month), 12 (± 1 month), and 18 months (± 1 month). In this subset, cumulative BP load was recalculated using only these four time points to ensure comparability of measurement intervals across individuals. Finally, to assess the potential impact of variability in BP measurement devices, we conducted a sensitivity analysis restricted to a small subset of participants who measured BP using the OMRON HBP-9031C device.

## Results

### Baseline characteristics

Between June 2017 and July 2024, a total of 33,872 participants with type 2 diabetes had a follow-up duration of at least 1.5 years. After applying the inclusion and exclusion criteria, 18,168 participants were included in the final analysis—13,388 for arterial stiffness progression analysis and 11,145 for DKD incidence analysis. Baseline characteristics of participants included in the analysis versus those excluded are presented in Table S3. Throughout the study period, 131,973 blood pressure measurements were recorded, with each participant having an average of six measurements. We visualized the number of BP measurements of all participants using frequency histograms (Figure S3). The distribution of cumulative BP load, calculated based on these blood pressure measurements from the two cohorts, is shown in Figures S4 and S5.

Table 1 presents the baseline characteristics of the study participants. The participants had a mean (SD) age of 54.5 (11.0) years, and 10,453 (57.5%) were male. During a mean follow-up period of 3.5 years, the median annual increase in ba-PWV was 0.19 m/s. Among the participants analyzed, arterial stiffness progression occurred in 6,366 (47.6% of 13,388), and incident DKD occurred in 2,855 (25.6% of 11,145). Participants with arterial stiffness progression were older and had a longer duration of diabetes compared to those without progression. They also exhibited a higher prevalence of cardiovascular disease and hypertension. Similarly, participants who developed DKD were older and had a longer duration of diabetes. They also exhibited a higher prevalence of cardiovascular disease and were more likely to have a history of hypertension compared to those who did not develop DKD.

**Table 1:** Baseline characteristics of participants stratified by arterial stiffness progression and the incidence of diabetic kidney disease

	Overall	Progression of arterial stiffness	No Progression of arterial stiffness	*P*	Overall	Diabetic kidney disease	No diabetic kidney disease	*P*
No. of participants	13,388	6,366	7,022		11,145	2,855	8,290	
Age, years	54.83 ± 10.96	58.51 ± 9.62	51.50 ± 11.03	< 0.001	53.46 ± 10.93	55.83 ± 10.81	52.65 ± 10.85	< 0.001
Males, n (%)	7,555 (56.43%)	3,324 (52.21%)	4,231 (60.25%)	< 0.001	6,655 (59.71%)	1,491 (52.22%)	5,164 (62.29%)	< 0.001
Duration of diabetes, years	7.68 ± 7.00	9.07 ± 7.27	6.43 ± 6.50	< 0.001	6.14 ± 6.51	7.39 ± 7.00	5.71 ± 6.28	< 0.001
History of CVD, %	1,825 (13.68%)	1,062 (16.75%)	763 (10.90%)	< 0.001	1,277 (11.54%)	411 (14.49%)	866 (10.53%)	< 0.001
History of hypertension, %	5,869 (44.40%)	3,247 (51.73%)	2,622 (37.78%)	< 0.001	3,851 (35.03%)	1,262 (45.01%)	2,589 (31.61%)	< 0.001
Education level, n (%)				< 0.001				< 0.001
Less than high school	7,819 (58.55%)	3,840 (60.46%)	3,979 (56.81%)		5,531 (49.93%)	1,675 (59.06%)	3,856 (46.79%)	
High school or further	5,536 (41.45%)	2,511 (39.54%)	3,025 (43.19%)		5,546 (50.07%)	1,161 (40.94%)	4,385 (53.21%)	
Occupation				0.014				< 0.001
Intermediate or low grade	9,526 (71.30%)	4,594 (72.31%)	4,932 (70.39%)		7,494 (67.56%)	2,085 (73.42%)	5,409 (65.55%)	
High grade	3,834 (28.70%)	1,759 (27.69%)	2,075 (29.61%)		3,598 (32.44%)	755 (26.58%)	2,843 (34.45%)	
Annual Household Income, thousand CNY				< 0.001				< 0.001
< 10	445 (3.57%)	249 (4.15%)	196 (3.04%)		365 (3.56%)	105 (3.92%)	260 (3.44%)	
10–30	1,812 (14.56%)	1,000 (16.66%)	812 (12.60%)		1,336 (13.04%)	412 (15.38%)	924 (12.22%)	
31–100	4,860 (39.04%)	2,270 (37.81%)	2,590 (40.19%)		3,904 (38.12%)	1,065 (39.75%)	2,839 (37.54%)	
101–300	3,186 (25.59%)	1,357 (22.60%)	1,829 (28.38%)		2,842 (27.75%)	683 (25.49%)	2,159 (28.55%)	
> 300	2,146 (17.24%)	1,128 (18.79%)	1,018 (15.80%)		1,795 (17.53%)	414 (15.45%)	1,381 (18.26%)	
Diet score	2.48 ± 0.98	2.48 ± 1.00	2.48 ± 0.96	0.831	2.56 ± 0.99	2.53 ± 0.98	2.57 ± 0.99	0.060
Diet score category				0.003				0.066
0–1	2,160 (17.27%)	1,097 (18.30%)	1,063 (16.33%)		1,529 (14.95%)	408 (15.41%)	1,121 (14.79%)	
2–3	8,442 (67.51%)	3,961 (66.06%)	4,481 (68.85%)		6,904 (67.52%)	1,814 (68.53%)	5,090 (67.17%)	
4–5	1,902 (15.21%)	938 (15.64%)	964 (14.81%)		1,792 (17.53%)	425 (16.06%)	1,367 (18.04%)	
Physical activity at goal, n (%)				0.033				0.035
No	11,972 (90.16%)	5,732 (90.74%)	6,240 (89.63%)		9,630 (87.45%)	2,499 (88.59%)	7,131 (87.06%)	
Yes	1,307 (9.84%)	585 (9.26%)	722 (10.37%)		1,382 (12.55%)	322 (11.41%)	1,060 (12.94%)	
Systolic blood pressure, mmHg	132.05 ± 18.96	133.94 ± 19.51	130.35 ± 18.28	< 0.001	128.49 ± 16.68	131.29 ± 17.09	127.53 ± 16.43	< 0.001
Diastolic blood pressure, mmHg	76.67 ± 11.45	75.99 ± 11.58	77.28 ± 11.29	< 0.001	76.35 ± 10.66	76.15 ± 10.89	76.42 ± 10.58	0.253
HbA1c, %	8.37 ± 2.05	8.46 ± 2.05	8.28 ± 2.04	< 0.001	8.25 ± 2.05	8.33 ± 2.05	8.23 ± 2.05	0.019
HbA1c, mmol/mol	67.93 ± 22.37	68.99 ± 22.40	66.97 ± 22.30	< 0.001	66.69 ± 22.39	67.54 ± 22.40	66.39 ± 22.38	0.019
Body mass index, kg/m^2^	26.03 ± 3.69	25.94 ± 3.58	26.10 ± 3.78	0.009	25.54 ± 3.56	25.78 ± 3.50	25.46 ± 3.58	< 0.001
Triglycerides, mmol/L	2.13 ± 2.20	2.08 ± 2.08	2.18 ± 2.30	0.015	2.02 ± 1.95	2.02 ± 1.87	2.02 ± 1.98	0.985
Total cholesterol, mmol/L	4.94 ± 1.29	4.90 ± 1.30	4.98 ± 1.29	0.001	4.87 ± 1.21	4.87 ± 1.23	4.88 ± 1.21	0.785
HDL cholesterol, mmol/L	1.23 ± 0.34	1.24 ± 0.35	1.21 ± 0.33	< 0.001	1.21 ± 0.33	1.22 ± 0.33	1.21 ± 0.33	0.008
LDL cholesterol, mmol/L	2.92 ± 1.00	2.90 ± 1.03	2.93 ± 0.97	0.045	2.94 ± 0.96	2.92 ± 1.02	2.95 ± 0.94	0.186
eGFR, ml/min/1.73m^2^	98.11 ± 19.19	93.93 ± 18.91	101.90 ± 18.65	< 0.001	101.94 ± 14.53	99.61 ± 15.93	102.75 ± 13.93	< 0.001
UACR, mg/mmol	2.43 [1.04;5.85]	2.50 [1.22;7.03]	2.00 [0.90;4.90]	0.010	1.15 [0.70;1.97]	1.48 [0.90;2.30]	1.05 [0.65;1.78]	< 0.001
ba-PWV measurement, m/s	16.11 ± 3.24	17.7 ± 3.35	15.24 ± 2.88	< 0.001	15.35 ± 3.00	16.00 ± 3.23	15.12 ± 2.89	< 0.001
Antihypertensive agents, n (%)	4,517 (33.74%)	2,556 (40.15%)	1,961 (27.93%)	< 0.001	2,840 (25.48%)	925 (32.40%)	1,915 (23.10%)	< 0.001

Continuous variables are presented as mean ± standard deviation (SD) or median (interquartile range), while categorical variables are summarized as counts and percentages. Group differences were assessed using Student's t-test, Wilcoxon rank-sum test, or chi-square test

Abbreviations: CVD = cardiovascular diseases, HbA1c = glycated hemoglobin, eGFR = estimated glomerular filtration rate, UACR = urine albumin-to-creatinine ratio, ba-PWV = brachial-ankle pulse wave velocity

### Association between cumulative BP load and cardio-renal outcomes

As shown in Table 2, cumulative BP load was significantly associated with both the absolute and relative annual changes in ba-PWV. For the absolute change, the β coefficients and standard error (SE) were 0.239 (0.009), 0.201 (0.009), and 0.150 (0.008) when cumulative SBP load was calculated using targets of < 120 mmHg, < 130 mmHg, and < 140 mmHg, respectively. For the relative change, the corresponding β values were 0.014 (0.001), 0.012 (0.001), and 0.009 (0.001). No evidence of multicollinearity was observed, as all variance inflation factor values were < 2 across models.

**Table 2:** Associations between cumulative BP load and annual changes in ba-PWV with model performance evaluation

	β (SE)	*P* value	VIF	Adjusted R^2^ (95%CI)
Annual absolute change in ba-PWV				
*Cumulative SBP load*				
Target: SBP < 120 mmHg	0.239 (0.009)	< 0.001	1.97	0.193 (0.180–0.200)
Target: SBP < 130 mmHg	0.201 (0.009)	< 0.001	1.74	0.184 (0.169–0.191)
Target: SBP < 140 mmHg	0.150 (0.008)	< 0.001	1.51	0.172 (0.158–0.180)
Cumulative DBP load				
Target: DBP < 80 mmHg	0.143 (0.009)	< 0.001	1.59	0.170 (0.155–0.175)
Target: DBP < 90 mmHg	0.077 (0.008)	< 0.001	1.23	0.159 (0.145–0.165)
Annual relative change in ba-PWV				
*Cumulative SBP load*				
Target: SBP < 120 mmHg	0.014 (0.001)	< 0.001	1.97	0.200 (0.188–0.207)
Target: SBP < 130 mmHg	0.012 (0.001)	< 0.001	1.74	0.192 (0.180–0.198)
Target: SBP < 140 mmHg	0.009 (0.001)	< 0.001	1.51	0.182 (0.170–0.189)
*Cumulative DBP load*				
Target: DBP < 80 mmHg	0.009 (0.0005)	< 0.001	1.59	0.182 (0.168–0.185)
Target: DBP < 90 mmHg	0.005 (0.0005)	< 0.001	1.23	0.171 (0.158–0.175)

The β coefficients and p values were adjusted for baseline covariates including age, sex, diabetes duration, blood pressure, HbA1c, BMI, triglycerides, total cholesterol, high-density lipoprotein cholesterol, low-density lipoprotein cholesterol, history of cardiovascular disease, history of hypertension, and use of antihypertensive medications. In addition, the number of BP measurements and baseline ba-PWV were also included

Abbreviations: SE = standard error; VIF = variance inflation factor

**Fig. 2:**
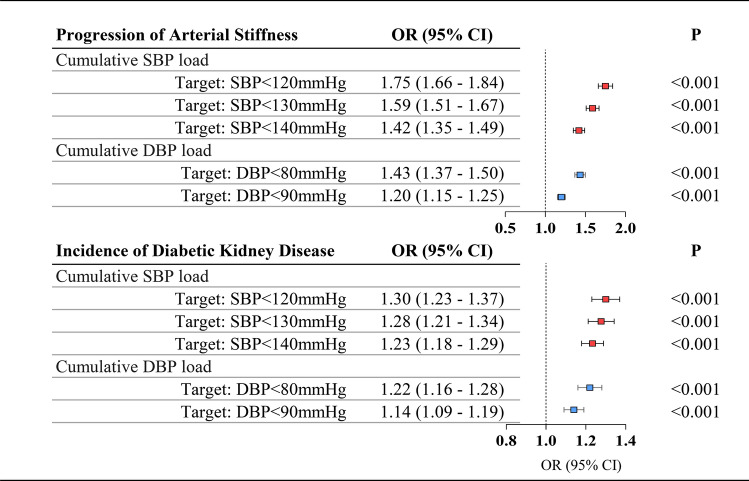
Associations of cumulative BP load with arterial stiffness progression and incident diabetic kidney disease. The odds ratios (OR) and 95% confidence intervals (CI) were adjusted for baseline covariates including age, sex, diabetes duration, blood pressure, HbA1c, BMI, triglycerides, total cholesterol, high-density lipoprotein cholesterol, low-density lipoprotein cholesterol, history of cardiovascular disease, history of hypertension, and use of antihypertensive medications. In addition, the number of BP measurements was included. Analyses for arterial stiffness progression were additionally adjusted for baseline ba-PWV, while models for incident DKD were adjusted for baseline eGFR

Figure 2 presents the results of logistic regression models evaluating the associations between cumulative BP load and two binary outcomes: progression of arterial stiffness and incidence of DKD, using different clinically recommended BP targets. After adjusting for potential confounding factors, each SD increase in cumulative SBP load was associated with higher odds of arterial stiffness progression, with odds ratios (OR) of 1.75 (95% CI 1.66–1.84), 1.59 (1.51–1.67), and 1.42 (1.35–1.49) for SBP targets of < 120 mmHg, < 130 mmHg, and < 140 mmHg, respectively. For the incidence of DKD, the corresponding ORs were 1.30 (95% CI 1.23–1.37), 1.28 (1.21–1.34), and 1.23 (1.18–1.29), respectively.

**Fig. 3:**
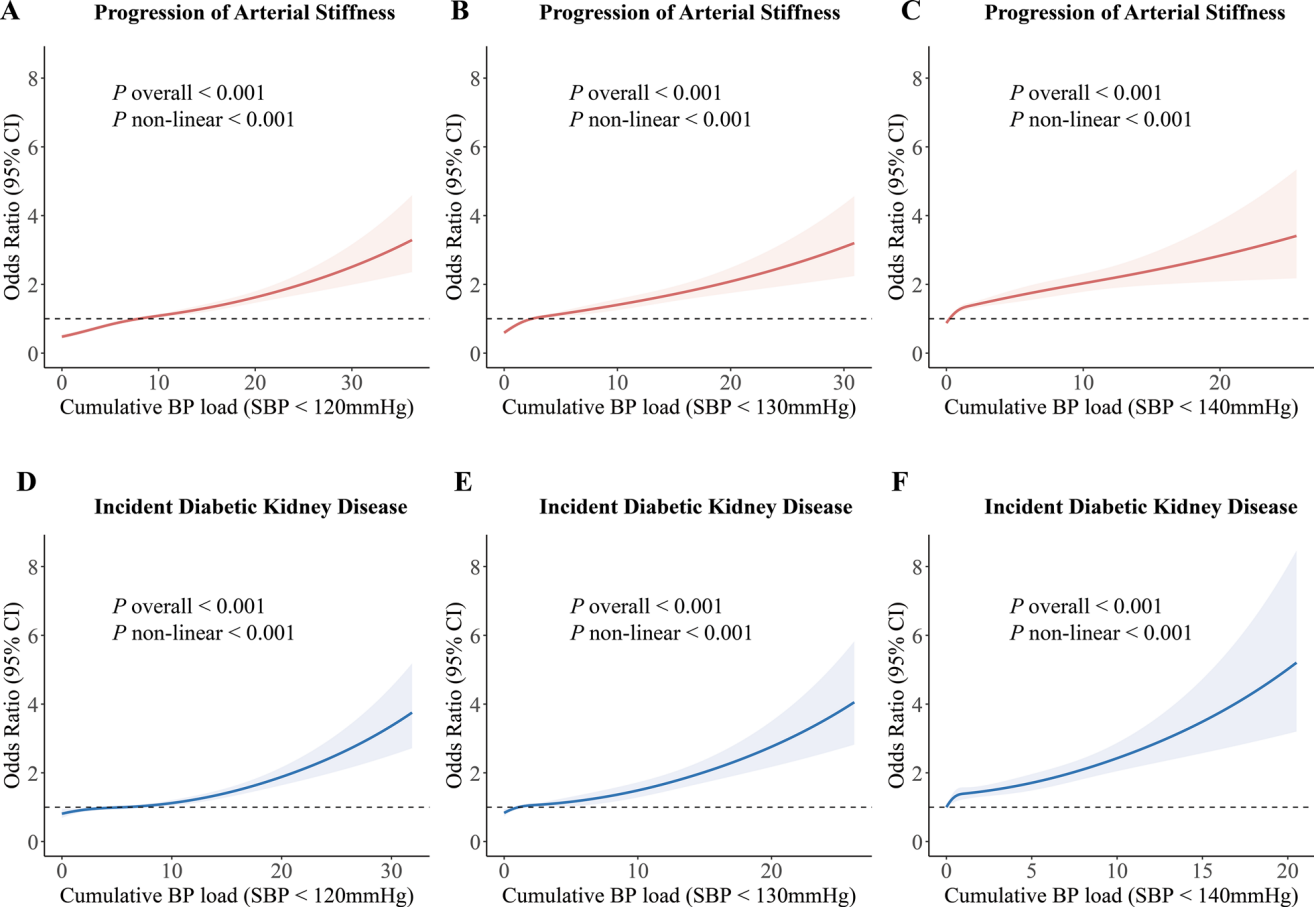
Restricted cubic spline curves for the associations of cumulative SBP load with arterial stiffness progression (**A**–**C**) and incident DKD (**D**–**F**). Cumulative SBP loads were calculated based on target SBP < 120 mmHg, SBP < 130 mmHg, and SBP < 140 mmHg. All models were adjusted for baseline covariates including age, sex, diabetes duration, SBP, HbA1c, BMI, triglycerides, total cholesterol, high-density lipoprotein cholesterol, low-density lipoprotein cholesterol, history of cardiovascular disease, history of hypertension, and use of antihypertensive medications. The number of BP measurements was also adjusted. Analyses for arterial stiffness progression were additionally adjusted for baseline ba-PWV, while models for incident DKD were adjusted for baseline eGFR. Solid lines represent multivariable-adjusted odds ratios (ORs), and shaded areas indicate 95% confidence intervals (CIs). Knots for the cumulative BP load were placed at the 5th, 35th, 65th, and 95th percentiles. The Wald test was used to calculate the P values for nonlinearity

Restricted cubic spline analyses revealed dose–response relationships between cumulative BP load and cardio-renal outcomes (Fig. 3 and Figure S6). When cumulative SBP load (target SBP < 120 mmHg) exceeds 10, arterial stiffness progression risks and DKD incidence increase. As cumulative SBP load continues to rise, the rate of risk increase accelerates, demonstrating a typical J-shaped association. At other BP target values, cumulative BP load also demonstrated nonlinear associations with cardio-renal outcomes (all nonlinear *P* < 0.05).

### Prognostic value of cumulative BP load calculated based on varying targets

For the absolute annual change in ba-PWV, cumulative BP load calculated using a target SBP of < 120 mmHg was associated with the highest adjusted R^2^ (0.193; 95% CI 0.180–0.200) among the target levels tested. Adjusted R^2^ values for targets of < 130 mmHg and < 140 mmHg were 0.184 (95% CI 0.169–0.191) and 0.172 (95% CI 0.158–0.180), respectively. A similar pattern was observed for the relative annual change in ba-PWV, where the target of < 120 mmHg again yielded the highest adjusted R^2^ (0.200; 95% CI 0.188–0.207) among all SBP targets tested, followed by < 130 mmHg (0.192; 95% CI 0.180–0.198) and < 140 mmHg (0.182; 95% CI 0.170–0.189) (Table 2). In models predicting arterial stiffness progression, the incorporation of cumulative BP load based on a target of < 120 mmHg increased the C-statistic from 0.699 (base model) to 0.726 (Table 3). Targets of < 130 mmHg and < 140 mmHg yielded C-statistics of 0.720 and 0.713, respectively. Relative to the base model with traditional risk factors, the addition of cumulative BP load calculated using a target of < 120 mmHg was associated with a continuous NRI of 36.8% (95% CI 33.4–40.1%). NRIs for the < 130 mmHg and < 140 mmHg targets were 28.2% (95% CI 24.8–31.5%) and 16.2% (95% CI 12.9–19.5%), respectively.

**Table 3:** Prognostic value of cumulative BP load compared with traditional risk factors

	Akaike information criterion	C-Statistic (95% CI)	Continuous net reclassification improvement
Progression of arterial stiffness			
Base SBP model^*^	16,889	0.699 (0.690–0.708)	Reference
*Base model* + *cumulative SBP load*			
Target: SBP < 120 mmHg	16,398	0.726 (0.718–0.735)	36.8% (33.4–40.1%)
Target: SBP < 130 mmHg	16,526	0.720 (0.712–0.729)	28.2% (24.8–31.5%)
Target: SBP < 140 mmHg	16,668	0.713 (0.704–0.722)	16.2% (12.9–19.5%)
Base DBP model^*^	16,896	0.698 (0.690–0.707)	Reference
*Base model* + *cumulative DBP load*			
Target: DBP < 80 mmHg	16,675	0.711 (0.702–0.719)	28.1% (24.8–31.4%)
Target: DBP < 90 mmHg	16,823	0.702 (0.694–0.711)	22.1% (18.9–25.3%)
Incidence of diabetic kidney disease			
Base SBP model^*^	12,076	0.653 (0.642–0.665)	Reference
*Base model* + *cumulative SBP load*			
Target: SBP < 120 mmHg	11,987	0.662 (0.650–0.674)	15.8% (11.5–20.0%)
Target: SBP < 130 mmHg	11,989	0.661 (0.650–0.673)	12.5% (8.3–16.7%)
Target: SBP < 140 mmHg	11,997	0.660 (0.649–0.672)	6.4% (2.3–10.6%)
Base DBP model^*^	12,290	0.624 (0.612–0.636)	Reference
*Base model* + *cumulative DBP load*			
Target: DBP < 80 mmHg	12,228	0.632 (0.620–0.644)	12.8% (8.6–16.9%)
Target: DBP < 90 mmHg	12,255	0.629 (0.617–0.641)	9.6% (5.5–13.6%)

*Base SBP model contained baseline SBP and other traditional risk factors, including age, sex, diabetes duration, HbA1c, body mass index, triglycerides, total cholesterol, high-density lipoprotein cholesterol, low-density lipoprotein cholesterol, history of cardiovascular disease, history of hypertension, and use of antihypertensive medications

*Base DBP model contained baseline DBP and other traditional risk factors, including age, sex, diabetes duration, HbA1c, body mass index, triglycerides, total cholesterol, high-density lipoprotein cholesterol, low-density lipoprotein cholesterol, history of cardiovascular disease, history of hypertension, and use of antihypertensive medications

In addition to the common baseline covariates included in the models, baseline ba-PWV was additionally included in the model for arterial stiffness progression, and baseline eGFR was included in the model for incident DKD

Abbreviations: SBP = systolic blood pressure, DBP = diastolic blood pressure

For predicting the incidence of DKD, the cumulative BP load calculated based on a target SBP of < 120 mmHg showed a slightly better predictive performance compared to targets of < 130 mmHg and < 140 mmHg. When cumulative SBP loads, based on target SBP values of < 120 mmHg, < 130 mmHg, and < 140 mmHg, were incorporated into the base model, the C-statistic improved from 0.653 to 0.662, 0.661, and 0.660, respectively (Table 3). The NRIs for cumulative SBP load were 15.8% (95% CI 11.5–20.0%), 12.5% (8.3–16.7%), and 6.4% (2.3–10.6%), respectively.

For target DBP < 80 mmHg, cumulative DBP load demonstrated superior predictive ability for cardio-renal outcomes compared to a target DBP < 90 mmHg.

### Structural equation model for the progression of arterial stiffness and the incidence of DKD

**Fig. 4:**
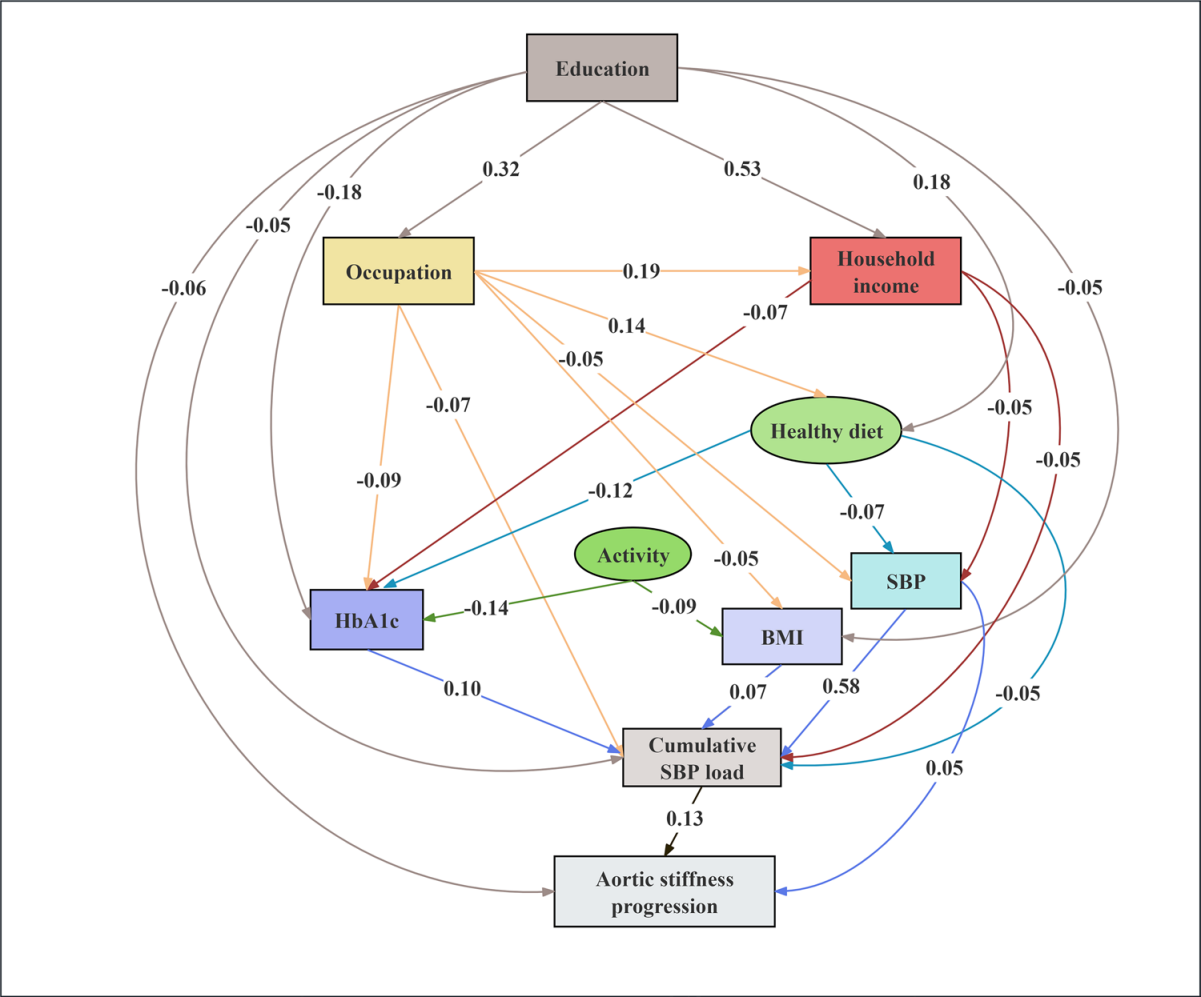
Structural-equation modeling for the pathways through cumulative SBP load to aortic stiffness progression. The cumulative SBP load was calculated using a target SBP of < 120 mmHg. Arrows indicate the direction of regression paths, with the numbers on the paths representing standardized path coefficients. Observable variables are shown as rectangles, and latent variables are shown as ellipses. Only statistically significant paths (*P* < 0.05) with path coefficients ≥ 0.05 are shown for simplicity. The model is adjusted for age and sex.

**Fig. 5:**
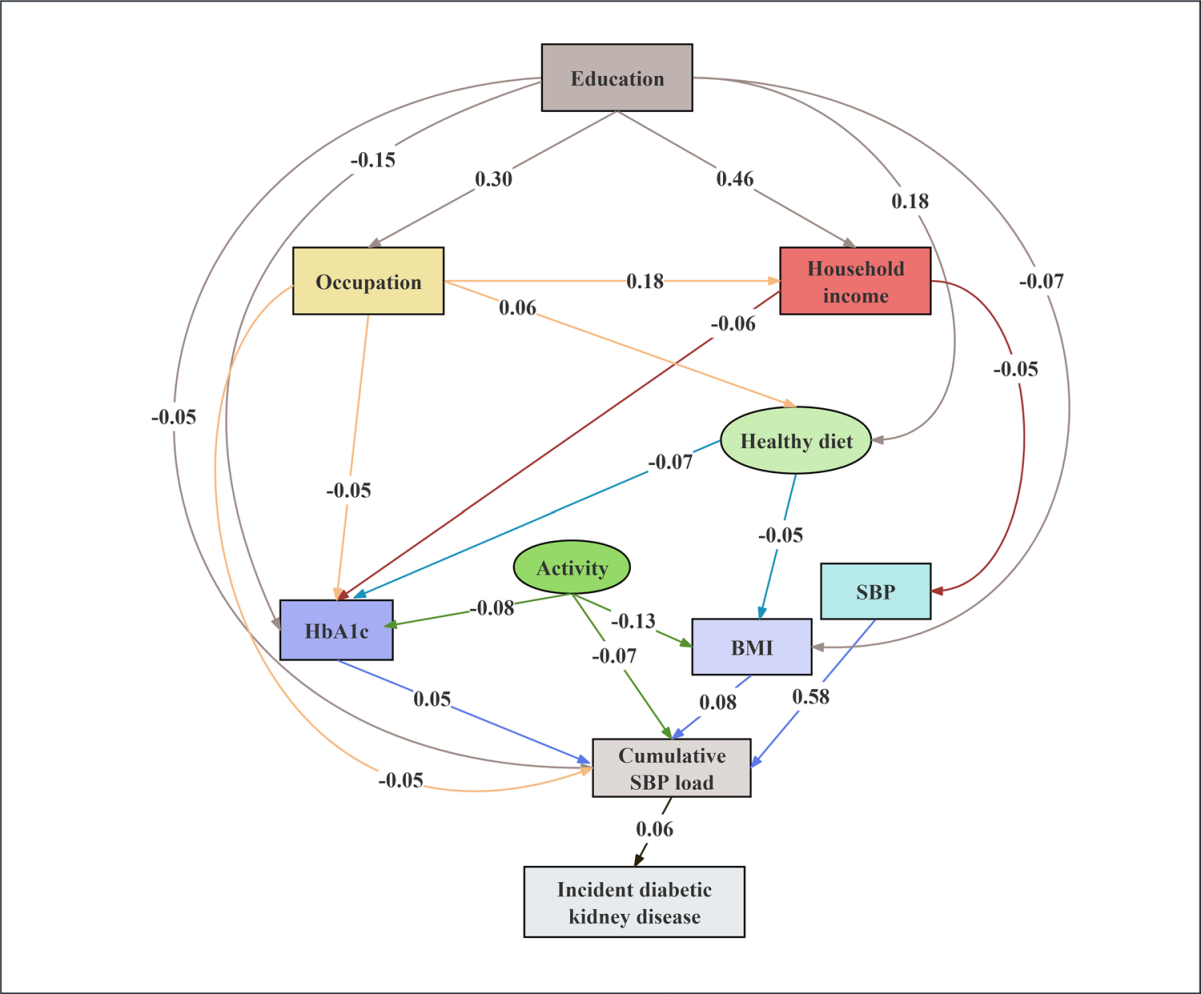
Structural-equation modeling for the pathways through cumulative SBP load to incident diabetic kidney disease. The cumulative SBP load was calculated using a target SBP of < 120 mmHg. Arrows indicate the direction of regression paths, with the numbers on the paths representing standardized path coefficients. Observable variables are shown as rectangles, and latent variables are shown as ellipses. Only statistically significant paths (*P* < 0.05) with path coefficients ≥ 0.05 are shown for simplicity. The model is adjusted for age and sex.

Structural equation modeling analyses demonstrated good model fits for both outcomes. Specifically, for arterial stiffness progression, the model showed good fit indices (RMSEA = 0.037 [SD = 0.007], CFI = 0.998 [SD = 0.001]) and an adequate TLI (0.929 [SD = 0.025]). Similarly, the DKD incidence model exhibited a good fit according to RMSEA (0.031 [SD = 0.005]) and CFI (0.996 [SD = 0.001]), with an adequate TLI (0.937 [SD = 0.021]) (Figs. 4 and 5, Tables S4-5).

Higher educational level (β_AS_ = − 0.05, β_DKD_ = − 0.05), better occupation status (β_AS_ = − 0.07, β_DKD_ = − 0.05), and higher annual household income (β_AS_ = − 0.05, β_DKD_ = − 0.03) were significantly correlated with lower cumulative SBP load across both outcomes. Conversely, higher baseline HbA1c (β_AS_ = 0.10, β_DKD_ = 0.05), BMI (β_AS_ = 0.07, β_DKD_ = 0.08), and SBP (β_AS_ = 0.58, β_DKD_ = 0.58) were associated with increased cumulative SBP load. Healthier dietary habits also showed significant relationships with reduced cumulative SBP load in both models (β_AS_ = − 0.05, β_DKD_ = − 0.04). Achieving physical activity goals was significantly associated with lower cumulative SBP load only in the DKD incidence model (β_DKD_ = − 0.07), but this relationship was not significant in the arterial stiffness progression model. Detailed statistical results, including confidence intervals for each pathway, are available in Supplementary Tables S4-5.

Sensitivity analyses showed consistent results after excluding participants with a history of cardiovascular diseases (Tables S6-7). In subgroup analyses stratified by baseline hypertension status, the cumulative SBP load based on a control target of < 120 mmHg consistently showed the highest predictive performance among all BP targets tested in both hypertensive and non-hypertensive patients. Similarly, cumulative DBP load calculated using a control target of < 80 mmHg performed best across both subgroups (Tables S8-11). Furthermore, BP TITRE and BP variability were independently associated with the progression of arterial stiffness and the incidence of DKD. However, the predictive performance of BP TITRE and BP variability was inferior to that of cumulative BP load calculated using the predefined control targets (SBP < 120 mmHg and DBP < 80 mmHg), as indicated by lower C-statistics and NRI values (Tables S12-13). In the subsample of participants with BP measurements recorded at baseline, 6 (± 1 month), 12 (± 1 month), and 18 (± 1 month) months, cumulative SBP load was recalculated using only these four time points. The SBP < 120 mmHg-based cumulative BP load again demonstrated the highest predictive performance among all BP targets tested for both arterial stiffness progression and incident DKD in this subset, consistent with the findings from the main analysis (Tables S14-15). In the subsample of participants whose BP was measured using the OMRON HBP-9031C device, the cumulative BP load based on the target of SBP < 120 mmHg demonstrated the highest predictive performance among all BP targets tested, for both arterial stiffness progression and incident DKD (Tables S16–S17).

## Discussion

In this large-scale longitudinal study, we compared the predictive value of cumulative BP load calculated using different blood pressure target values for the progression of arterial stiffness and the incidence of DKD in patients with type 2 diabetes. Our results demonstrated that cumulative BP load with a target of SBP < 120 mmHg exhibited the most potent predictive capability. Structural equation modeling indicated that socioeconomic factors, including higher education, better occupation, and higher income, were associated with lower cumulative SBP load, potentially through correlations with healthier dietary habits and improved metabolic parameters, which in turn correlated with better cardio-renal outcomes.

Hypertension has been well-established as a shared risk factor for cardiovascular and chronic kidney disease [46, 47]. A comprehensive meta-analysis involving 344,716 adults demonstrated that each five mmHg increase in SBP was associated with a 10% higher risk of cardiovascular diseases, a 13% higher risk of stroke, an 8% higher risk of ischemic heart disease, and a 5% higher risk of cardiovascular mortality [48]. Similarly, findings from a large community-based hypertension cohort study revealed that the risk of chronic renal failure progressively increased when SBP exceeded 120 mmHg, with every 10 mmHg rise in SBP correlating to a 6% greater risk of CKD [49]. While these studies provide strong evidence linking elevated SBP to cardiovascular and renal outcomes, their reliance on single-point blood pressure measurements limits understanding the cumulative impact of blood pressure on target organ damage.

In previous studies, both cumulative BP and cumulative BP load have been used to quantify long-term blood pressure exposure. Cumulative BP refers to the product of blood pressure and time (measured in mmHg × year), which continuously accumulates over the observation period, failing to account for variations in BP [11]. Cumulative BP load reflects BP elevations by calculating the proportion of the blood pressure–time area under the curve exceeding the target value relative to the total area under the curve. Once a patient's blood pressure is effectively controlled, cumulative BP load decreases accordingly. Therefore, cumulative BP load can sensitively identify high-risk individuals with elevated blood pressure and evaluate the effectiveness of blood pressure interventions.

Recent studies have highlighted the strong association between cumulative BP load and adverse cardiovascular and renal outcomes. Wang et al. demonstrated that elevated cumulative SBP load (target SBP < 130 mmHg) significantly increases cardiovascular disease risk in patients with type 2 diabetes [16]. Arterial stiffness is a recognized precursor to cardiovascular diseases. Our findings confirmed a significant association between increased cumulative BP load and arterial stiffness progression. Additionally, Park et al. have shown that cumulative SBP load (target SBP < 120 mmHg) is strongly linked to an elevated risk of chronic kidney disease [15]. Given the inconsistencies in target definitions of cumulative SBP load across studies above, we systematically compared the predictive value of cumulative SBP load under three different control targets (SBP < 140 mmHg, < 130 mmHg, and < 120 mmHg). Our results indicated that cumulative SBP load with a target of < 120 mmHg demonstrated superior predictive performance, evidenced by improved C-statistics, the highest adjusted R^2^ and NRI values among all BP targets tested. We also compared the predictive value of cumulative DBP load under two different control targets (DBP < 90 mmHg and DBP < 80 mmHg), finding that DBP < 80 mmHg performed better. Although our findings indicate that cumulative SBP load based on a target of < 120 mmHg demonstrated the highest predictive performance, this should not be interpreted as a recommendation for intensive BP lowering for all patients in routine clinical practice. Evidence from prior trials has shown that while more intensive SBP control can improve certain outcomes, it is also associated with an increased risk of hypotension [6, 50]. Therefore, the adoption of lower SBP targets should be individualized, with close monitoring to ensure safety and tolerability—particularly among elderly patients, individuals in poor health, or those with established target organ damage or severe coronary artery disease.

Moreover, our restricted cubic spline analysis revealed that once cumulative SBP load (target SBP < 120 mmHg) exceeded 10, the risks of arterial stiffness progression and diabetic kidney disease increased. Notably, calculating cumulative BP load requires only routine follow-up data, incurring no additional costs. Thus, we propose that electronic health record (EHR) systems integrate a function to compute cumulative SBP load, similar to body mass index. This feature could trigger timely alerts when a patient's cumulative SBP load exceeds 10, allowing clinicians to intervene early and implement appropriate measures.

To identify potential targets for reducing cumulative BP load, we used structural equation modeling to investigate the pathways linking modifiable risk factors—including socioeconomic status, lifestyle behaviors, and metabolic indicators—with cumulative SBP load, arterial stiffness progression, and DKD incidence.

Our results indicated that higher educational attainment was linked to better occupational status and higher household income, factors associated with improved blood pressure management. Bin et al. reported that individuals with higher educational attainment and income are more likely to access emerging technologies, such as innovative blood pressure management applications and wearable devices, which can lead to more effective blood pressure management [51]. Metabolic control, as reflected by lower baseline HbA1c and BMI, was significantly associated with reduced cumulative SBP load, reinforcing the importance of metabolic management in diabetes care. In both outcome models, healthier dietary habits showed consistent associations with lower cumulative SBP load. However, the relationship between physical activity and cumulative SBP load varied between the two outcomes. Specifically, physical activity was inversely associated with cumulative SBP load in the DKD incidence model, whereas this association was not statistically significant in the arterial stiffness progression model. This discrepancy may partly reflect the methodological characteristics inherent in structural equation modeling. Structural equation modeling simultaneously accounts for multiple correlated pathways and covariates, potentially attenuating or masking certain direct associations observable in simpler regression models [52, 53]. In the arterial stiffness progression model, metabolic factors such as HbA1c showed stronger associations and thus may have explained a substantial portion of the variance previously linked with physical activity. Additionally, the self-reported nature of physical activity measurement could introduce potential biases, possibly influencing the observed associations. Future studies employing objective assessment methods, such as accelerometers or multiple-sensor devices, are warranted to clarify the precise relationships among physical activity, cumulative SBP load, and metabolic indicators concerning arterial stiffness progression.

## Strengths and limitations

This study has several notable strengths. First, it is based on a large-scale, multicenter longitudinal cohort of diabetes patients, with over 100,000 blood pressure measurements, ensuring robust statistical power. Second, we systematically compared the predictive value of cumulative BP load based on varying targets. Third, we applied structural equation modeling to comprehensively evaluate the associations among socioeconomic factors (education, occupation, and income), health behaviors, metabolic indicators, and cumulative blood pressure load across different clinical outcomes.

However, this study has several limitations. First, all blood pressure measurements were obtained using cuff-based devices. Cuff-based devices can be affected by body movement, positional changes, and device-specific algorithms used to interpret oscillometric signals [54–56]. To reduce these potential inaccuracies, our study employed BP monitors from a uniform manufacturer (Omron Healthcare), adhered to standardized measurement protocols, and ensured that all personnel underwent stringent training to minimize measurement errors. Second, BP data were based exclusively on clinic measurements. While office BP is commonly used for the diagnosis, classification, and management of hypertension, it does not fully capture short-term fluctuations or nighttime BP patterns [57]. Future studies should incorporate out-of-office BP assessments, such as ambulatory blood pressure monitoring (ABPM) and home blood pressure monitoring (HBPM), to better capture short-term BP dynamics and improve the accuracy of long-term BP exposure estimation. Third, this study utilized peripheral blood pressure measurements instead of central blood pressure (cBP). Considering that cBP may more accurately reflect the hemodynamic burden on target organs, future research should further investigate its potential clinical utility [58]. Fourth, the follow-up duration was relatively short, which limited our ability to assess the long-term impact of cumulative BP load on cardiovascular outcomes. Finally, residual confounding may exist due to unmeasured factors, including genetic predisposition and social determinants such as neighborhood conditions, housing, and access to care.

## Conclusions

This study demonstrates that cumulative BP load with the target of SBP < 120 mmHg has a superior predictive ability for the progression of arterial stiffness and incidence of DKD compared to traditional single office BP measurements. Structural equation modeling further revealed that favorable socioeconomic factors—including higher education level, occupational status, and household income—were associated with reduced cumulative SBP load through positive correlations with healthy lifestyle behaviors (dietary habits) and improved metabolic indicators (BMI, HbA1c). A lower cumulative SBP load, in turn, was associated with delayed onset of cardio-renal complications. These findings highlight the importance of monitoring long-term cumulative BP load in diabetes management.

## Supplementary Information

Supplementary Material

## Data Availability

The datasets generated and/or analyzed during the current study are not publicly available but are available from the corresponding author on reasonable request.

## Abbreviations

ABPM: Ambulatory blood pressure monitoring
AIC: Akaike information criterion
ARV: Average real variability
AUC: Area under the curve
ba-PWV: Brachial-ankle pulse wave velocity
BMI: Body mass index
BP: Blood pressure
cBP: Central blood pressure
CFI: Comparative fit index
CI: Confidence interval
CVD: Cardiovascular disease
DBP: Diastolic blood pressure
DKD: Diabetic kidney disease
eGFR: Estimated glomerular filtration rate
HbA1c: Glycated hemoglobin
HBPM: Home blood pressure monitoring
MICE: Multiple imputations by chained equations
MMC: Metabolic Management Center
NRI: Net reclassification improvement
OR: Odds ratios
RCS: Restricted cubic splines
RMSA: Root mean square error of approximation
SBP: Systolic blood pressure
SD: Standard deviation
SE: Standard error
STROBE: Strengthening the reporting of observational studies in epidemiology
TITRE: Time at target
TLI: Tucker-Lewis index
UACR: Urine albumin-to-creatinine ratio
VIF: Variance inflation factor
