# Supplementary material for: Cumulative blood pressure load as a predictor of arterial stiffness progression and incident diabetic kidney disease: a multicenter longitudinal study
Source: Cardiovasc Diabetol. 2025 May 24;24:225. doi: 10.1186/s12933-025-02785-7 (PMC12103019; doi:10.1186/s12933-025-02785-7)
Supplement: Supplementary file 1 — Supplementary Material [file 12933_2025_2785_MOESM1_ESM.docx]

Supplementary material

Cumulative Blood Pressure Load as a Predictor of Arterial Stiffness Progression and Incident Diabetic Kidney Disease: A Multicenter Longitudinal Study

**Table of Contents**

[Supplementary Methods 3](#_Toc194577127)

[Measurement and definitions for modifiable factors 3](#_Toc194577128)

[Figure S1: Flow chart and inclusion criteria for the arterial stiffness progression cohort and the diabetic kidney disease cohort 4](#_Toc194577129)

[Figure S2: the Definition of Arterial Stiffness Progression Outcomes 5](#_Toc194577131)

[Figure S3: Distributions of the number of BP measurements in arterial stiffness progression analysis (n=13,388) and incident diabetic kidney disease analysis (n=11,145) 6](#_Toc194577132)

[Figure S4: Distributions of cumulative BP load in arterial stiffness progression analysis (n=13,388) 7](#_Toc194577133)

[Figure S5: Distributions of cumulative BP load in diabetic kidney disease analysis (n=11,145) 8](#_Toc194577134)

[Figure S6. Restricted cubic spline (RCS) curves for the associations of cumulative DBP load with the progression of arterial stiffness (A, B) and diabetic kidney disease (C, D). 9](#_Toc194577135)

[Table S1. Completeness of recording for characteristics of the participants at baseline 10](#_Toc194577137)

[Table S2. Criteria for Selecting Risk Factors for the Structural Equation Modeling 11](#_Toc194577138)

[Table S3. Baseline Characteristics of Participants Included vs. Excluded from the Analysis 12](#_Toc194577139)

[Table S4. Standardized path coefficients with bias-corrected bootstrap confidence intervals for the structural equation model in the arterial stiffness progression analysis (n = 13,388) 13](#_Toc194577140)

[Table S5. Standardized path coefficients with bias-corrected bootstrap confidence intervals for the structural equation model in diabetic kidney disease analysis (n = 11,145) 14](#_Toc194577141)

[Table S6. Associations of cumulative BP load with progression of arterial stiffness in patients without CVD (n = 11,515) 15](#_Toc194577142)

[Table S7. Prognostic value of cumulative BP load compared with traditional risk factors in patients without CVD (n = 11,515) 16](#_Toc194577143)

[Table S8. Associations of cumulative BP load with the progression of arterial stiffness (n = 5,869) and DKD (n = 3,851) in patients with a history of hypertension 17](#_Toc194577144)

[Table S9. Prognostic value of cumulative BP load compared with traditional risk factors in patients with a history of hypertension 18](#_Toc194577145)

[Table S10. Associations of cumulative BP load with the progression of arterial stiffness (n = 7,349) and DKD (n = 7,144) in patients without a history of hypertension 19](#_Toc194577146)

[Table S11. Prognostic value of cumulative BP load compared with traditional risk factors in patients without a history of hypertension 20](#_Toc194577147)

[Table S12. Associations of BP TITRE and BP variability with progression of arterial stiffness and diabetic kidney disease 21](#_Toc194577148)

[Table S13. Prognostic Value of BP TITRE and BP Variability Compared with Traditional Risk Factors 22](#_Toc194577149)

[Table S14. Associations of cumulative BP load with the progression of arterial stiffness (n = 2,012) and DKD (n = 1,968) in patients with four fixed BP measurements 23](#_Toc194577150)

[Table S15. Prognostic value of cumulative BP load compared with traditional risk factors in patients with four fixed BP measurements 24](#_Toc194577151)

[Table S16. Associations of cumulative BP load with the progression of arterial stiffness (n = 2,243) and DKD (n = 2,037) in patients whose BP was measured using the OMRON HBP-9031C device 25](#_Toc194577152)

[Table S17. Prognostic value of cumulative BP load compared with traditional risk factors in patients whose BP was measured using the OMRON HBP-9031C device 26](#_Toc194577153)

[Table S18. STROBE Statement—Checklist of items that should be included in reports of cohort studies 27](#_Toc194577154)

[Reference 29](#_Toc194577155)

Supplementary Methods

Measurement and definitions for modifiable factors

Socioeconomic status was assessed using three indicators: education level, occupational status, and annual household income. Education level was classified as follows: less than high school (≤9 years) and high school or further (>9 years). Occupation was categorized into intermediate/low-grade (support staff, service workers, unemployed, or retired individuals) and high-grade (executive, administrative, or professional positions). Annual household income was categorized into <10, 10-30, 31-100, 101-300, and >300 thousand CNY. Dietary intake was assessed using a food questionnaire to capture habitual dietary patterns. A composite healthy diet score (range: 0-5) was calculated based on five components: daily vegetable/fruit intake (≥4.5 cups), weekly fish consumption (≥2 servings, ≥3.5-oz/serving), daily soy protein intake (≥25g), daily sodium intake (<1500mg), and weekly sugar-sweetened beverage consumption (≤450 kcal). Each component meeting the criteria contributed one point to the total score [1]. Physical activity was assessed using the short form of the International Physical Activity Questionnaire (IPAQ), based on self-reported frequency and average daily duration of vigorous- and moderate-intensity activities at leisure time. Participants were classified as meeting the physical activity goal if they engaged in ≥75 minutes of vigorous activity, ≥150 minutes of moderate activity, or an equivalent combination per week [2].

Figure S1: Flow chart and inclusion criteria for the arterial stiffness progression cohort and the diabetic kidney disease cohort


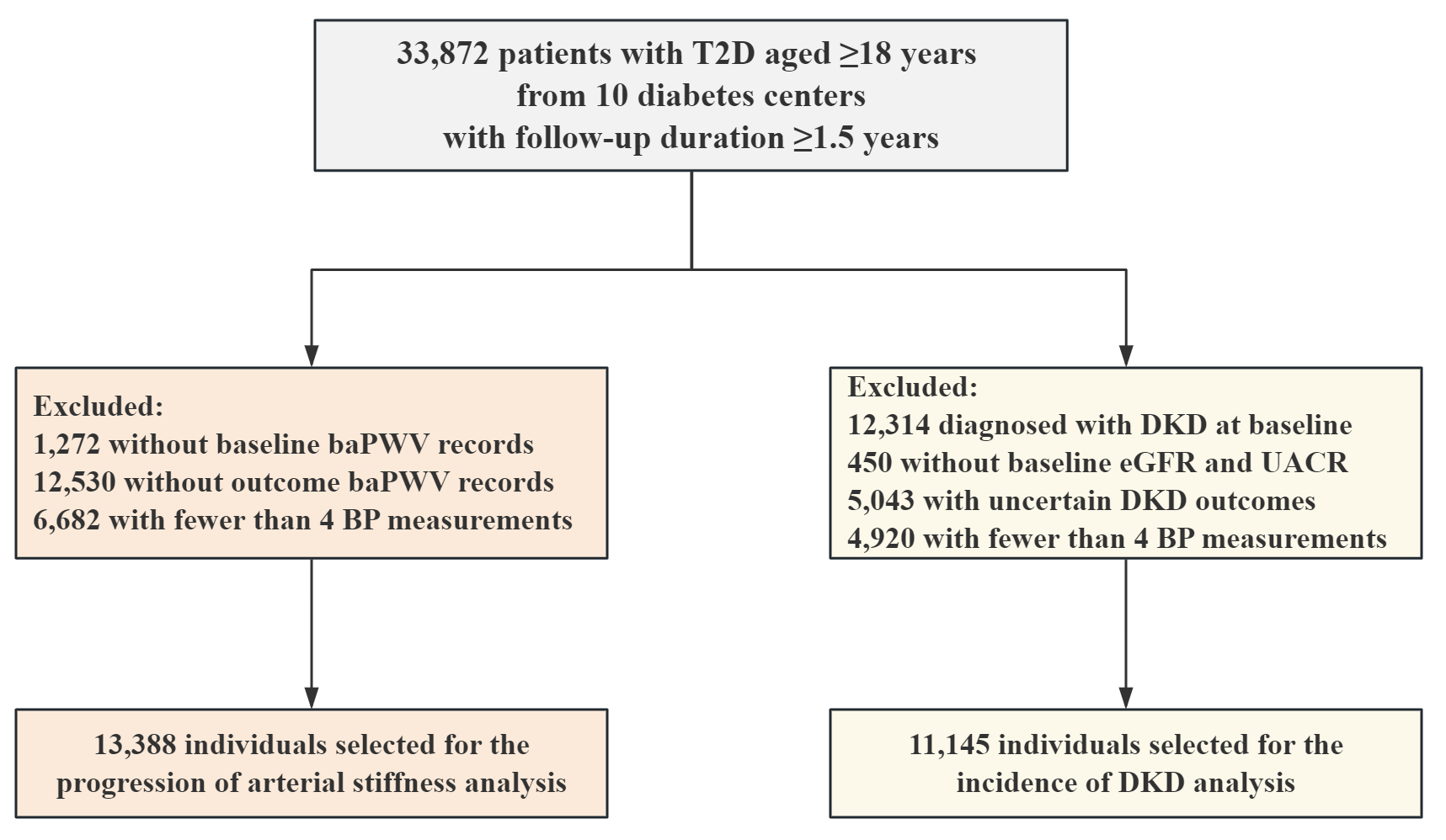


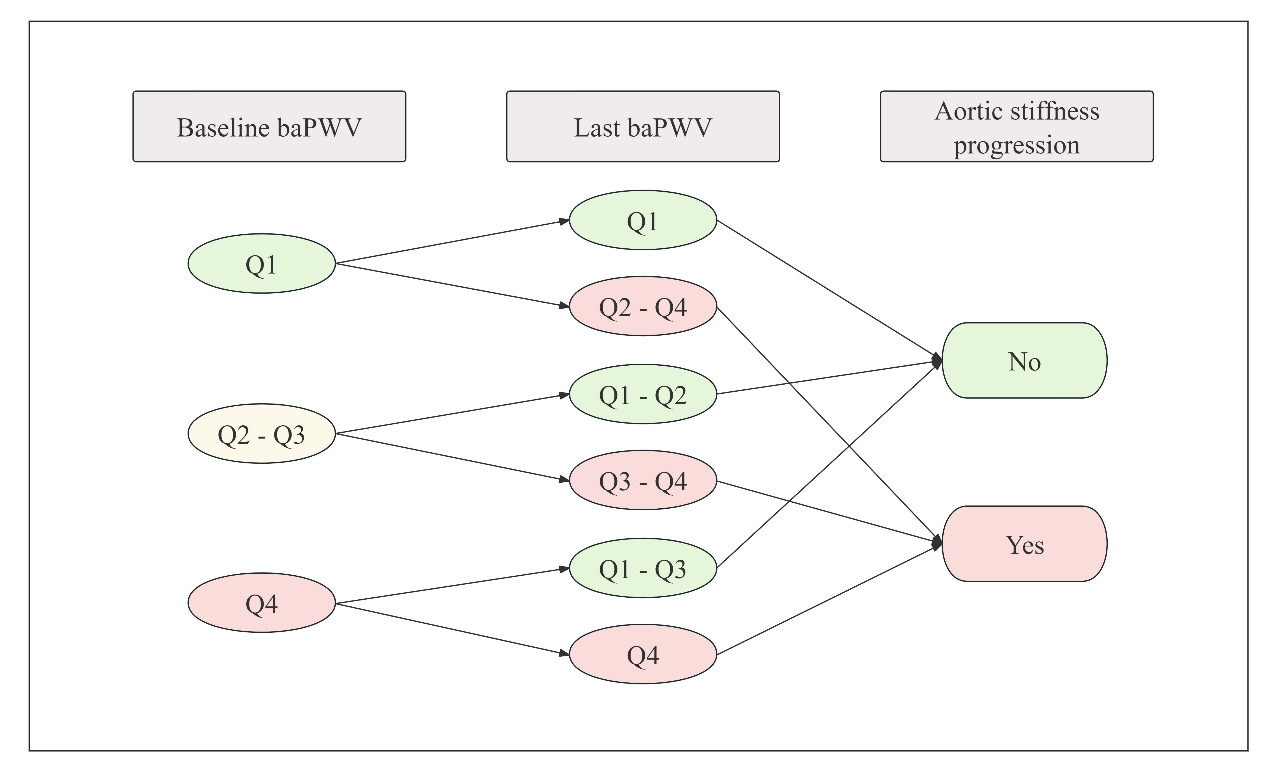
Figure S2: the Definition of Arterial Stiffness Progression Outcomes

Figure S3: Distributions of the number of BP measurements in arterial stiffness progression analysis (n=13,388) and incident diabetic kidney disease analysis (n=11,145)


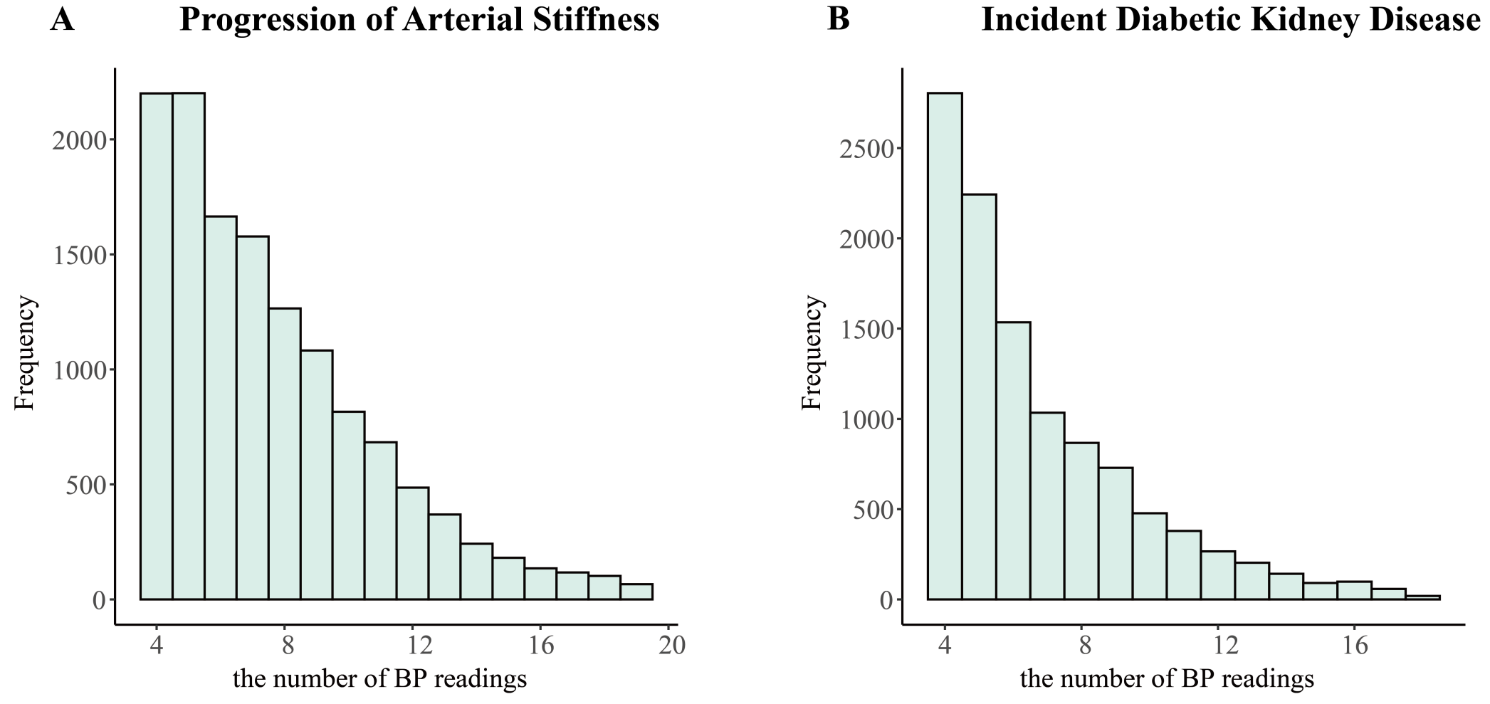


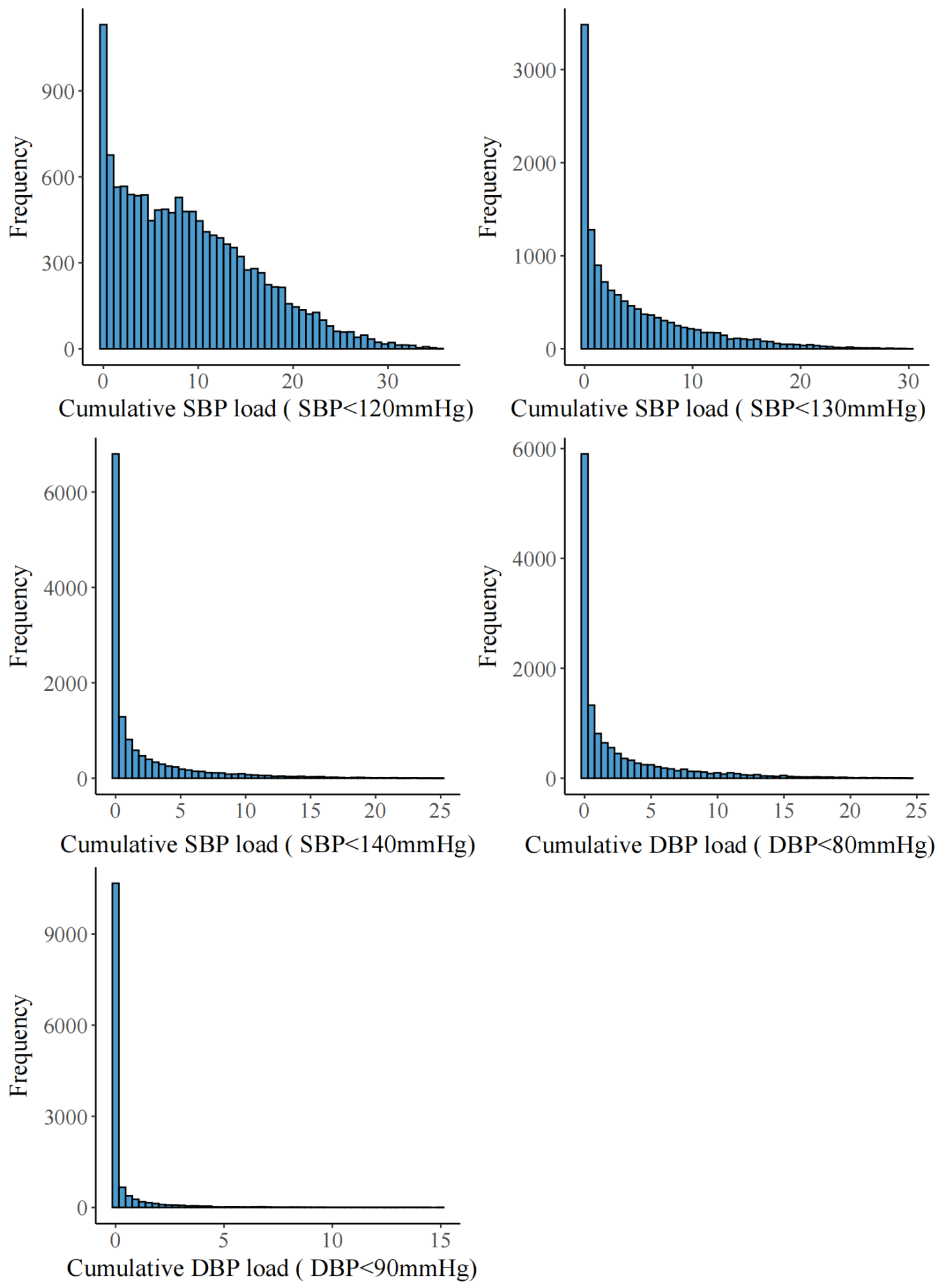
Figure S4: Distributions of cumulative BP load in arterial stiffness progression analysis (n=13,388)


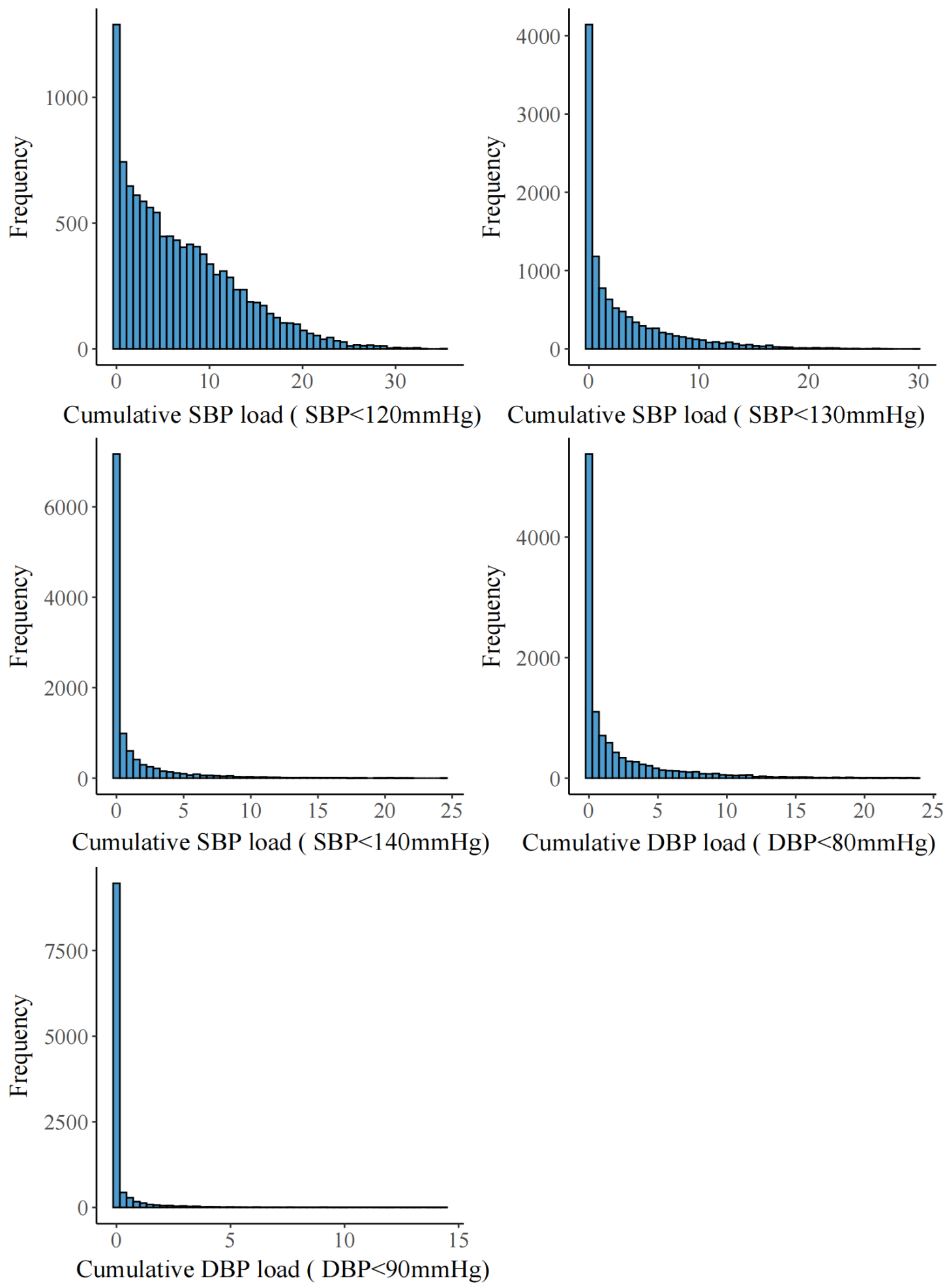
Figure S5: Distributions of cumulative BP load in diabetic kidney disease analysis (n=11,145)

Figure S6. Restricted cubic spline (RCS) curves for the associations of cumulative DBP load with the progression of arterial stiffness (A, B) and diabetic kidney disease (C, D).


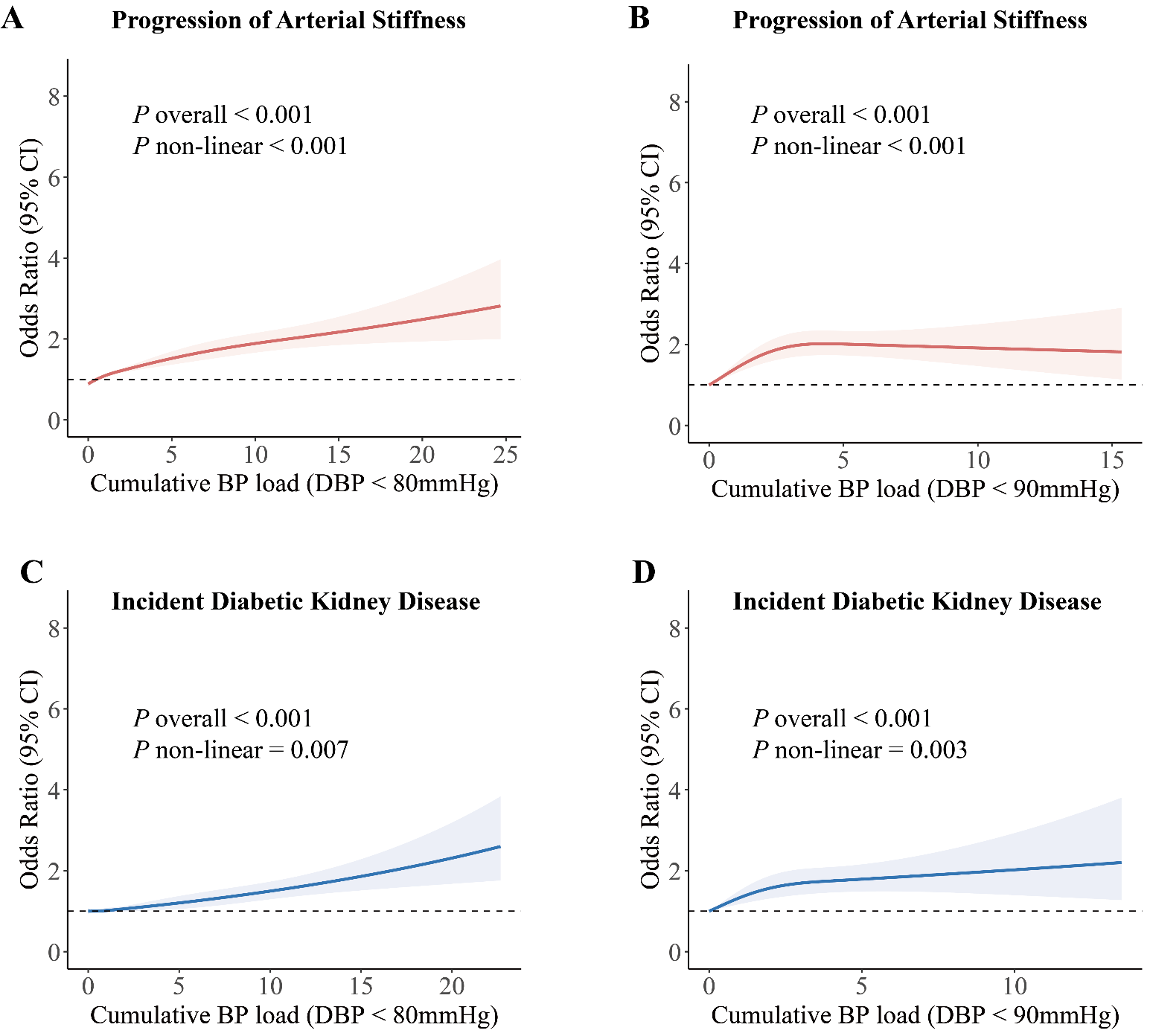


The cumulative DBP load was calculated based on target DBP <80 mmHg and DBP <90 mmHg. All models were adjusted for baseline covariates including age, sex, diabetes duration, DBP, HbA1c, BMI, triglycerides, total cholesterol, high-density lipoprotein cholesterol, low-density lipoprotein cholesterol, history of cardiovascular disease, history of hypertension, and use of antihypertensive medications. The number of BP measurements was also adjusted. Analyses for arterial stiffness progression were additionally adjusted for baseline ba-PWV, while models for incident DKD were adjusted for baseline eGFR. Solid lines represent multivariable-adjusted odds ratios (ORs), and shaded areas indicate 95% confidence intervals (CIs). Knots for the cumulative BP load were placed at the 5th, 35th, 65th, and 95th percentiles. The Wald chi-square test was used to calculate the P values for nonlinearity.

| Table S1. Completeness of recording for characteristics of the participants at baseline | | | | |
| --- | --- | --- | --- | --- |
|  | **Progression of**  **Arterial Stiffness (n=13,388)** | **Incident Diabetic Kidney Disease (n=11,145)** | |  |
| Age | 13,388 (100) | | 11,145 (100) | |
| Sex | 13,388 (100) | | 11,145 (100) | |
| Duration of diabetes | 13,360 (99.79) | | 11,104 (99.63) | |
| History of CVD | 13,340 (99.64) | | 11,063 (99.26) | |
| History of hypertension | 13,218 (98.73) | | 10,995 (98.65) | |
| Education level | 13,355 (99.75) | | 11,077 (99.39) | |
| Occupation | 13,360 (99.79) | | 11,092 (99.52) | |
| Annual Household Income | 12,449 (92.99) | | 10,242 (91.90) | |
| Diet score | 12,504 (93.40) | | 10,225 (91.75) | |
| Physical activity at goal | 13,279 (99.19) | | 11,012 (98.81) | |
| Systolic blood pressure | 13,388 (100) | | 11,145 (100) | |
| Diastolic blood pressure | 13,388 (100) | | 11,145 (100) | |
| HbA1c | 13,324 (99.52) | | 11,124 (99.81) | |
| Body mass index | 13,364 (99.82) | | 11,091 (99.52) | |
| Triglycerides | 13,235 (98.86) | | 11,110 (99.69) | |
| Total cholesterol | 13,207 (98.65) | | 11,094 (99.54) | |
| HDL cholesterol | 13,091 (97.78) | | 11,023 (98.91) | |
| LDL cholesterol | 13,187 (98.50) | | 11,076 (99.38) | |
| eGFR | 13,207 (98.65) | | 11,145 (100) | |
| UACR | 12,761 (95.32) | | 11,145 (100) | |
| ba-PWV measurement | 13,388 (100) | | 10,934 (98.11) | |
| Antihypertensive agents | 13,061 (97.56) | | 10,784 (96.76) | |

Abbreviations: CVD = cardiovascular diseases, HbA1c = glycated hemoglobin, eGFR = estimated glomerular filtration rate, UACR = urine albumin-to-creatinine ratio, ba-PWV = brachial-ankle pulse wave velocity.

Table S2. Criteria for Selecting Risk Factors for the Structural Equation Modeling

| **Criterion** | **Description** |
| --- | --- |
| 1. Biological plausibility | Supported by prior studies and pathophysiological evidence |
| 2. Statistical association | Demonstrated association with diabetic complications in previous research |
| 3. Prevalence | Exposure rate ≥10% among study participants |
| 4. Modifiability | Risk factors are clinically modifiable |
| 5. Data availability | Variables were available and reliably measurable in our database |
| 6. Missing data | Proportion of missing values <10% to ensure data integrity |

Table S3. Baseline Characteristics of Participants Included vs. Excluded from the Analysis

|  |  | **Progression of Arterial Stiffness** | | |  | **Incident Diabetic Kidney Disease** | | |
| --- | --- | --- | --- | --- | --- | --- | --- | --- |
|  |  | **Excluded** | **Included** | **P** |  | **Excluded** | **Included** | **P** |
| No. of participants |  | 20,484 | 13,388 |  |  | 22,727 | 11,145 |  |
| Age, years |  | 54.84 ± 11.32 | 54.83 ± 10.96 | 0.762 |  | 55.10 ± 11.67 | 53.46 ± 10.93 | <0.001 |
| Males, n (%) |  | 11162 (57.79%) | 7555 (56.43%) | 0.015 |  | 13236 (56.35%) | 6655 (59.71%) | <0.001 |
| Duration of diabetes, years |  | 6.93 ± 7.08 | 7.68 ± 7.00 | <0.001 |  | 7.56 ± 7.23 | 6.14 ± 6.51 | <0.001 |
| History of CVD, % |  | 2683 (14.23%) | 1825 (13.68%) | 0.166 |  | 3393 (14.78%) | 1277 (11.54%) | <0.001 |
| History of hypertension, % |  | 8137 (43.34%) | 5869 (44.40%) | 0.065 |  | 10714 (46.93%) | 3851 (35.03%) | <0.001 |
| Systolic blood pressure, mmHg |  | 132.44 ± 18.35 | 132.05 ± 18.96 | 0.080 |  | 133.77 ± 19.19 | 128.49 ± 16.68 | <0.001 |
| Diastolic blood pressure, mmHg |  | 78.29 ± 11.54 | 76.67 ± 11.45 | <0.001 |  | 78.18 ± 11.87 | 76.35 ± 10.66 | <0.001 |
| HbA1c, % |  | 8.51 ± 1.93 | 8.37 ± 2.05 | <0.001 |  | 8.75 ± 2.19 | 8.25 ± 2.05 | <0.001 |
| Body mass index, kg/m^2^ |  | 26.10 ± 3.85 | 26.03 ± 3.69 | 0.102 |  | 26.28 ± 3.93 | 25.54 ± 3.56 | <0.001 |
| Triglycerides, mmol/L |  | 2.23 ± 2.33 | 2.13 ± 2.20 | <0.001 |  | 2.28 ± 2.45 | 2.02 ± 1.95 | <0.001 |
| Total cholesterol, mmol/L |  | 4.96 ± 1.32 | 4.94 ± 1.29 | 0.426 |  | 5.00 ± 1.36 | 4.87 ± 1.21 | <0.001 |
| HDL cholesterol, mmol/L |  | 1.17 ± 0.33 | 1.23 ± 0.34 | <0.001 |  | 1.19 ± 0.34 | 1.21 ± 0.33 | <0.001 |
| LDL cholesterol, mmol/L |  | 2.99 ± 0.99 | 2.92 ± 1.00 | <0.001 |  | 2.98 ± 1.01 | 2.94 ± 0.96 | 0.006 |

Continuous variables are presented as mean ± standard deviation (SD) or median (interquartile range), while categorical variables are summarized as counts and percentages. Group differences were assessed using Student's t-test, Wilcoxon rank-sum test, or chi-square test.

Abbreviations: CVD = cardiovascular diseases, HbA1c = glycated hemoglobin.

Table S4. Standardized path coefficients with bias-corrected bootstrap confidence intervals for the structural equation model in the arterial stiffness progression
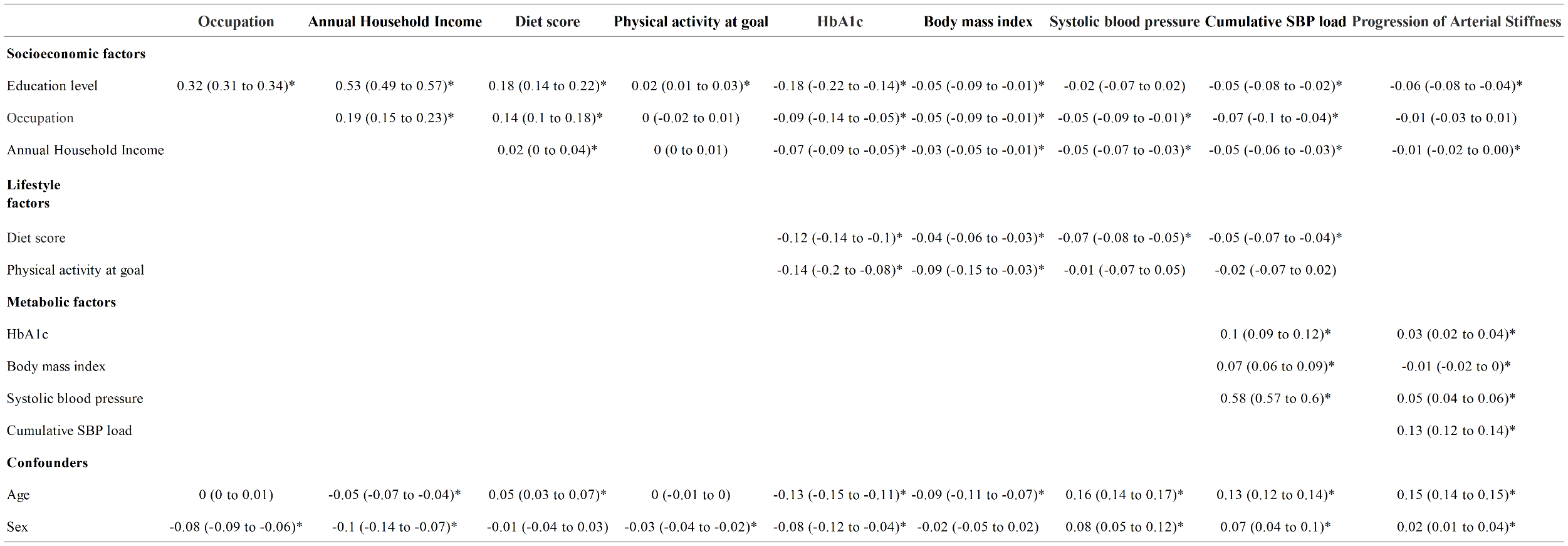
 analysis (n = 13,388)

RMSEA = 0.037 (SD = 0.007); CFI = 0.998 (SD = 0.001); TLI = 0.929 (SD = 0.025).

Abbreviations: HbA1c = glycated hemoglobin.

**
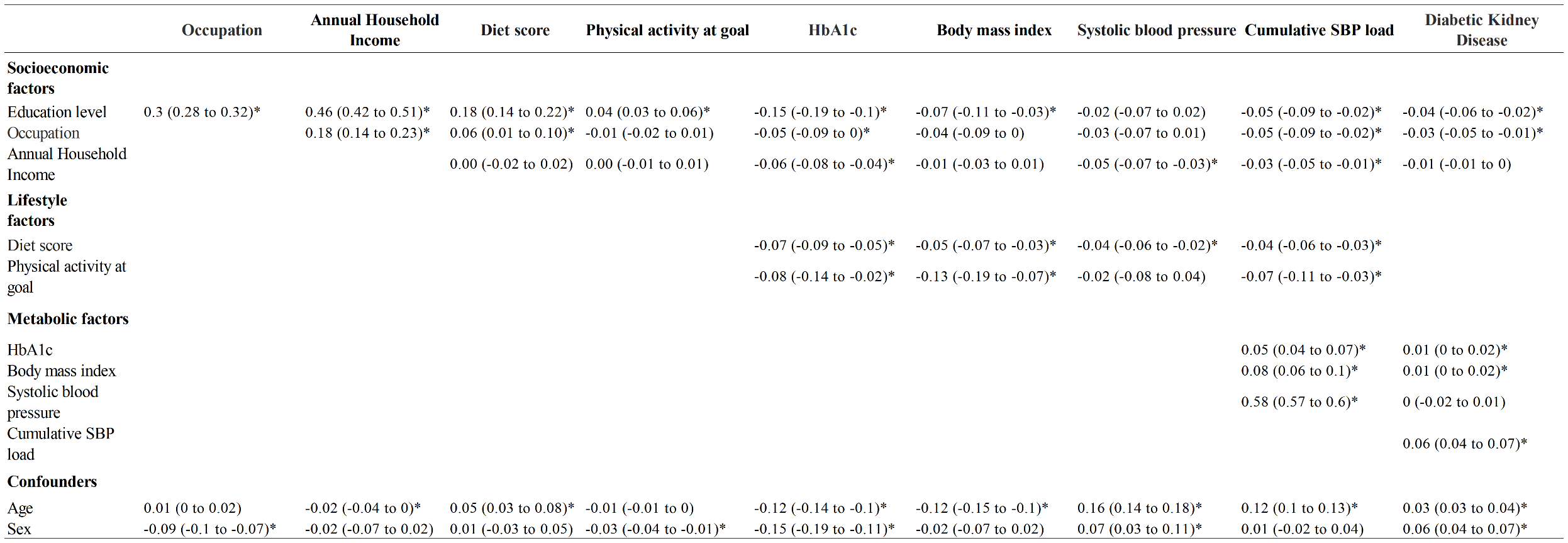
**Table S5. Standardized path coefficients with bias-corrected bootstrap confidence intervals for the structural equation model in Incident diabetic kidney disease analysis (n = 11,145)

RMSEA = 0.031 (SD = 0.005), CFI = 0.996 (SD = 0.001) indicated a good fit, and TLI = 0.937 (SD = 0.021)

Abbreviations: HbA1c = glycated hemoglobin.

| Table S6. Associations of cumulative BP load with progression of arterial stiffness in patients without CVD (n = 11,515) | | | |
| --- | --- | --- | --- |
|  |  | **OR (95% CI)** | **P** |
| Cumulative SBP load |  |  |  |
| Target: SBP<120mmHg |  | 1.76 (1.67 - 1.87) | <0.001 |
| Target: SBP<130mmHg |  | 1.60 (1.52 - 1.69) | <0.001 |
| Target: SBP<140mmHg |  | 1.43 (1.35 - 1.51) | <0.001 |
| Cumulative DBP load |  |  |  |
| Target: DBP<80mmHg |  | 1.45 (1.38 - 1.52) | <0.001 |
| Target: DBP<90mmHg |  | 1.20 (1.15 - 1.25) | <0.001 |

The odds ratios (OR) and 95% confidence intervals (CI) were adjusted for baseline covariates including age, sex, diabetes duration, blood pressure, HbA1c, BMI, triglycerides, total cholesterol, high-density lipoprotein cholesterol, low-density lipoprotein cholesterol, history of hypertension, and use of antihypertensive medications. In addition, the number of BP measurements and baseline ba-PWV were also included.

Abbreviations: SBP = systolic blood pressure, DBP = diastolic blood pressure.

| Table S7. Prognostic value of cumulative BP load compared with traditional risk factors in patients without CVD (n = 11,515) | | | | | | |  |
| --- | --- | --- | --- | --- | --- | --- | --- |
|  | **Akaike Information Criterion** | | **C-Statistic  (95% CI)** | | **Continuous Net Reclassification Improvement** | |  |
| Base SBP model | | 14,531 | | 0.713 (0.704 - 0.722) | | Reference | |
| Base SBP model + cumulative SBP load | |  | |  | |  | |
| Target: SBP<120mmHg | | 14,097 | | 0.736 (0.727 - 0.745) | | 35.2% (31.6% - 38.8%) | |
| Target: SBP<130mmHg | | 14,210 | | 0.731 (0.722 - 0.740) | | 26.6% (23.0% - 30.2%) | |
| Target: SBP<140mmHg | | 14,337 | | 0.725 (0.716 - 0.734) | | 15.4% (11.8% - 19.0%) | |
| Base DBP model | | 14,533 | | 0.713 (0.704 - 0.723) | | Reference | |
| Base DBP model + cumulative DBP load | |  | |  | |  | |
| Target: DBP<80mmHg | | 14,326 | | 0.726 (0.717 - 0.735) | | 30.6% (27.1% - 34.2%) | |
| Target: DBP<90mmHg | | 14,466 | | 0.717 (0.708 - 0.727) | | 22.7% (19.2% - 26.2%) | |

*Base SBP model contained baseline SBP and other traditional risk factors, including age, sex, diabetes duration, HbA1c, body mass index, triglycerides, total cholesterol, high-density lipoprotein cholesterol, low-density lipoprotein cholesterol, history of hypertension, and use of antihypertensive medications.

*Base DBP model contained baseline DBP and other traditional risk factors, including age, sex, diabetes duration, HbA1c, body mass index, triglycerides, total cholesterol, high-density lipoprotein cholesterol, low-density lipoprotein cholesterol, history of hypertension, and use of antihypertensive medications.

In addition to the common baseline covariates included in the models, baseline ba-PWV was additionally included in the model for arterial stiffness progression, and baseline eGFR was included in the model for incident DKD.

Abbreviations: SBP = systolic blood pressure, DBP = diastolic blood pressure.

| Table S8. Associations of cumulative BP load with the progression of arterial stiffness (n = 5,869) and DKD (n = 3,851) in patients with a history of hypertension | | | |
| --- | --- | --- | --- |
|  |  | **OR (95% CI)** | **P** |
| **Progression of Arterial Stiffness** |  |  |  |
| Cumulative SBP load |  |  |  |
| Target: SBP<120mmHg |  | 1.63 (1.52 - 1.75) | <0.001 |
| Target: SBP<130mmHg |  | 1.54 (1.44 - 1.65) | <0.001 |
| Target: SBP<140mmHg |  | 1.41 (1.33 - 1.50) | <0.001 |
| Cumulative DBP load |  |  |  |
| Target: DBP<80mmHg |  | 1.52 (1.41 - 1.63) | <0.001 |
| Target: DBP<90mmHg |  | 1.23 (1.14 - 1.32) | <0.001 |
| **Incident Diabetic Kidney Disease** |  |  |  |
| Cumulative SBP load |  |  |  |
| Target: SBP<120mmHg |  | 1.30 (1.20 - 1.41) | <0.001 |
| Target: SBP<130mmHg |  | 1.27 (1.19 - 1.36) | <0.001 |
| Target: SBP<140mmHg |  | 1.24 (1.16 - 1.31) | <0.001 |
| Cumulative DBP load |  |  |  |
| Target: DBP<80mmHg |  | 1.22 (1.13 - 1.31) | <0.001 |
| Target: DBP<90mmHg |  | 1.15 (1.08 - 1.22) | <0.001 |

The odds ratios (OR) and 95% confidence intervals (CI) were adjusted for baseline covariates including age, sex, diabetes duration, blood pressure, HbA1c, BMI, triglycerides, total cholesterol, high-density lipoprotein cholesterol, low-density lipoprotein cholesterol, history of CVD, and use of antihypertensive medications. In addition, the number of BP measurements was included. Analyses for arterial stiffness progression were additionally adjusted for baseline ba-PWV, while models for incident DKD were adjusted for baseline eGFR.

| Table S9. Prognostic value of cumulative BP load compared with traditional risk factors in patients with a history of hypertension | | | |
| --- | --- | --- | --- |
|  | **Akaike Information Criterion** | **C-Statistic  (95% CI)** | **Continuous Net Reclassification Improvement** |
| **Progression of Arterial Stiffness** |  |  |  |
| Base SBP model | 7,638 | 0.661 (0.647 - 0.675) | Reference |
| Base SBP model + cumulative SBP load |  |  |  |
| Target: SBP<120mmHg | 7,444 | 0.689 (0.675 - 0.702) | 33.0% (27.9% - 38.1%) |
| Target: SBP<130mmHg | 7,456 | 0.688 (0.675 - 0.702) | 30.7% (25.7% - 35.7%) |
| Target: SBP<140mmHg | 7,500 | 0.683 (0.670 - 0.697) | 22.8% (17.9% - 27.8%) |
| Base DBP model | 7,632 | 0.662 (0.648 - 0.676) | Reference |
| Base DBP model + cumulative DBP load |  |  |  |
| Target: DBP<80mmHg | 7,549 | 0.675 (0.661 - 0.689) | 27.0% (22.0% - 32.0%) |
| Target: DBP<90mmHg | 7,596 | 0.666 (0.652 - 0.680) | 23.5% (18.6% - 28.4%) |
| **Incident Diabetic Kidney Disease** |  |  |  |
| Base SBP model | 4,747 | 0.624 (0.605 - 0.643) | Reference |
| Base SBP model + cumulative SBP load | 4,688 |  |  |
| Target: SBP<120mmHg | 4,754 | 0.639 (0.620 - 0.657) | 18.8% (12.2% - 25.5%) |
| Target: SBP<130mmHg | 4,694 | 0.639 (0.620 - 0.658) | 17.7% (11.1% - 24.3%) |
| Target: SBP<140mmHg | 4,689 | 0.638 (0.619 - 0.657) | 11.2% (4.7% - 17.7%) |
| Base DBP model | 4,754 | 0.617 (0.598 - 0.636) | Reference |
| Base DBP model + cumulative DBP load |  |  |  |
| Target: DBP<80mmHg | 4,725 | 0.628 (0.609 - 0.647) | 19.1% (12.7% - 25.6%) |
| Target: DBP<90mmHg | 4,733 | 0.625 (0.606 - 0.644) | 16.4% (9.8% - 23.0%) |

*Base SBP model contained baseline SBP and other traditional risk factors, including age, sex, diabetes duration, HbA1c, body mass index, triglycerides, total cholesterol, high-density lipoprotein cholesterol, low-density lipoprotein cholesterol, history of cardiovascular disease, and use of antihypertensive medications.

*Base DBP model contained baseline DBP and other traditional risk factors, including age, sex, diabetes duration, HbA1c, body mass index, triglycerides, total cholesterol, high-density lipoprotein cholesterol, low-density lipoprotein cholesterol, history of cardiovascular disease, and use of antihypertensive medications.

In addition to the common baseline covariates included in the models, baseline ba-PWV was additionally included in the model for arterial stiffness progression, and baseline eGFR was included in the model for incident DKD.

Abbreviations: SBP = systolic blood pressure, DBP = diastolic blood pressure.

| Table S10. Associations of cumulative BP load with the progression of arterial stiffness (n = 7,349) and DKD (n = 7,144) in patients without a history of hypertension | | | |
| --- | --- | --- | --- |
|  |  | **OR (95% CI)** | **P** |
| **Progression of Arterial Stiffness** |  |  |  |
| Cumulative SBP load |  |  |  |
| Target: SBP<120mmHg |  | 1.87 (1.73 - 2.03) | <0.001 |
| Target: SBP<130mmHg |  | 1.68 (1.54 - 1.82) | <0.001 |
| Target: SBP<140mmHg |  | 1.50 (1.37 - 1.64) | <0.001 |
| Cumulative DBP load |  |  |  |
| Target: DBP<80mmHg |  | 1.52 (1.41 - 1.63) | <0.001 |
| Target: DBP<90mmHg |  | 1.23 (1.14 - 1.32) | <0.001 |
| **Incident Diabetic Kidney Disease** |  |  |  |
| Cumulative SBP load |  |  |  |
| Target: SBP<120mmHg |  | 1.30 (1.20 - 1.41) | <0.001 |
| Target: SBP<130mmHg |  | 1.30 (1.20 - 1.40) | <0.001 |
| Target: SBP<140mmHg |  | 1.26 (1.16 - 1.36) | <0.001 |
| Cumulative DBP load |  |  |  |
| Target: DBP<80mmHg |  | 1.23 (1.15 - 1.32) | <0.001 |
| Target: DBP<90mmHg |  | 1.13 (1.06 - 1.21) | <0.001 |

The odds ratios (OR) and 95% confidence intervals (CI) were adjusted for baseline covariates including age, sex, diabetes duration, blood pressure, HbA1c, BMI, triglycerides, total cholesterol, high-density lipoprotein cholesterol, low-density lipoprotein cholesterol, and history of CVD. In addition, the number of BP measurements was included. Analyses for arterial stiffness progression were additionally adjusted for baseline ba-PWV, while models for incident DKD were adjusted for baseline eGFR.

| Table S11. Prognostic value of cumulative BP load compared with traditional risk factors in patients without a history of hypertension | | | |
| --- | --- | --- | --- |
|  | **Akaike Information Criterion** | **C-Statistic  (95% CI)** | **Continuous Net Reclassification Improvement** |
| **Progression of Arterial Stiffness** |  |  |  |
| Base SBP model | 9,027 | 0.706 (0.695 - 0.718) | Reference |
| Base SBP model + cumulative SBP load |  |  |  |
| Target: SBP<120mmHg | 8,732 | 0.737 (0.726 - 0.748) | 38.2% (33.7% - 42.8%) |
| Target: SBP<130mmHg | 8,837 | 0.727 (0.715 - 0.739) | 29.5% (24.9% - 34.0%) |
| Target: SBP<140mmHg | 8,927 | 0.718 (0.706 - 0.730) | 15.3% (10.9% - 19.7%) |
| Base DBP model | 9,039 | 0.705 (0.693 - 0.717) | Reference |
| Base DBP model + cumulative DBP load |  |  |  |
| Target: DBP<80mmHg | 8,902 | 0.720 (0.708 - 0.731) | 31.8% (27.3% - 36.3%) |
| Target: DBP<90mmHg | 9,004 | 0.709 (0.698 - 0.721) | 23.7% (19.3% - 28.1%) |
| **Incident Diabetic Kidney Disease** |  |  |  |
| Base SBP model | 7,338 | 0.598 (0.583 - 0.614) | Reference |
| Base SBP model + cumulative SBP load |  |  |  |
| Target: SBP<120mmHg | 7,295 | 0.611 (0.595 - 0.627) | 14.0% (8.4% - 19.5%) |
| Target: SBP<130mmHg | 7,295 | 0.609 (0.594 - 0.625) | 11.5% (6.0% - 17.0%) |
| Target: SBP<140mmHg | 7,306 | 0.607 (0.591 - 0.623) | 6.5% (1.1% - 12.0%) |
| Base DBP model | 7,350 | 0.594 (0.578 - 0.610) | Reference |
| Base DBP model + cumulative DBP load |  |  |  |
| Target: DBP<80mmHg | 7,316 | 0.603 (0.588 - 0.619) | 11.8% (6.3% - 17.3%) |
| Target: DBP<90mmHg | 7,336 | 0.599 (0.583 - 0.615) | 7.9% (2.5% - 13.2%) |

*Base SBP model contained baseline SBP and other traditional risk factors, including age, sex, diabetes duration, HbA1c, body mass index, triglycerides, total cholesterol, high-density lipoprotein cholesterol, low-density lipoprotein cholesterol, and history of cardiovascular disease.

*Base DBP model contained baseline DBP and other traditional risk factors, including age, sex, diabetes duration, HbA1c, body mass index, triglycerides, total cholesterol, high-density lipoprotein cholesterol, low-density lipoprotein cholesterol, and history of cardiovascular disease.

In addition to the common covariates included in the models, baseline ba-PWV was additionally included in the model for arterial stiffness progression, and baseline eGFR was included in the model for incident DKD.

Abbreviations: SBP = systolic blood pressure, DBP = diastolic blood pressure.

| Table S12. Associations of BP TITRE and BP variability with progression of arterial stiffness and diabetic kidney disease | | | | |
| --- | --- | --- | --- | --- |
| **Progression of Arterial Stiffness** |  | **OR (95% CI)** |  | **P** |
| SBP TITRE |  |  |  |  |
| Target: SBP<120mmHg |  | 0.66 (0.63 - 0.69) |  | <0.001 |
| Target: SBP<130mmHg |  | 0.63 (0.60 - 0.66) |  | <0.001 |
| Target: SBP<140mmHg |  | 0.67 (0.64 - 0.70) |  | <0.001 |
| SBP variability |  |  |  |  |
| SBP SD |  | 1.14 (1.10 - 1.19) |  | <0.001 |
| SBP ARV |  | 1.14 (1.10 - 1.19) |  | <0.001 |
| DBP TITRE |  |  |  |  |
| Target: DBP<80mmHg |  | 0.65 (0.62 - 0.69) |  | <0.001 |
| Target: DBP<90mmHg |  | 0.78 (0.75 - 0.82) |  | <0.001 |
| DBP variability |  |  |  |  |
| DBP SD |  | 1.02 (0.99 - 1.06) |  | 0.239 |
| DBP ARV |  | 1.03 (0.99 - 1.07) |  | 0.120 |
| **Incident Diabetic Kidney Disease** |  | **OR (95% CI)** |  | **P** |
| SBP TITRE |  |  |  |  |
| Target: SBP<120mmHg |  | 0.89 (0.85 - 0.94) |  | <0.001 |
| Target: SBP<130mmHg |  | 0.83 (0.78 - 0.87) |  | <0.001 |
| Target: SBP<140mmHg |  | 0.81 (0.77 - 0.85) |  | <0.001 |
| SBP variability |  |  |  |  |
| SBP SD |  | 1.11 (1.07 - 1.16) |  | <0.001 |
| SBP ARV |  | 1.10 (1.06 - 1.15) |  | <0.001 |
| DBP TITRE |  |  |  |  |
| Target: DBP<80mmHg |  | 0.87 (0.82 - 0.92) |  | <0.001 |
| Target: DBP<90mmHg |  | 0.86 (0.82 - 0.90) |  | <0.001 |
| DBP variability |  |  |  |  |
| DBP SD |  | 1.09 (1.05 - 1.14) |  | <0.001 |
| DBP ARV |  | 1.08 (1.03 - 1.12) |  | 0.001 |

The odds ratios (OR) and 95% confidence intervals (CI) were adjusted for baseline covariates including age, sex, diabetes duration, blood pressure, HbA1c, BMI, triglycerides, total cholesterol, high-density lipoprotein cholesterol, low-density lipoprotein cholesterol, history of cardiovascular disease, history of hypertension, and use of antihypertensive medications. In addition, the number of BP measurements was included. Analyses for arterial stiffness progression were additionally adjusted for baseline ba-PWV, while models for incident DKD were adjusted for baseline eGFR.

| Table S13. Prognostic Value of BP TITRE and BP Variability Compared with Traditional Risk Factors | | | |
| --- | --- | --- | --- |
|  | **Akaike Information Criterion** | **C-Statistic  (95% CI)** | **Continuous Net Reclassification Improvement** |
| **Progression of Arterial Stiffness** |  |  |  |
| Base SBP model | 16,889 | 0.699 (0.690 - 0.708) | Reference |
| Base model + SBP TITRE |  |  |  |
| Target: SBP<120mmHg | 16,569 | 0.716 (0.707 - 0.725) | 25.5% (22.1% - 28.8%) |
| Target: SBP<130mmHg | 16,555 | 0.717 (0.708 - 0.726) | 27.1% (23.8% - 30.4%) |
| Target: SBP<140mmHg | 16,528 | 0.718 (0.710 - 0.727) | 35.5% (32.1% - 38.8%) |
| Base model + SBP variability |  |  |  |
| SBP SD | 16,832 | 0.702 (0.693 - 0.711) | 11.6% (8.3% - 15.0%) |
| SBP ARV | 16,843 | 0.701 (0.692 - 0.710) | 10.9% (7.6% - 14.3%) |
| Base DBP model | 16,896 | 0.698 (0.690 - 0.707) | Reference |
| Base model + DBP TITRE |  |  |  |
| Target: DBP<90mmHg | 16,769 | 0.705 (0.696 - 0.714) | 25.9% (22.6% – 29.2%) |
| Target: DBP<80mmHg | 16,623 | 0.713 (0.704 - 0.722) | 25.2% (22.0% – 28.5%) |
| Base model + DBP variability |  |  |  |
| DBP SD | 16,895 | 0.698 (0.690 - 0.707) | 3.2% (0.6% - 6.5%) |
| DBP ARV | 16,896 | 0.698 (0.690 - 0.707) | 3.5% (0.1% - 6.8%) |
| **Incident Diabetic Kidney Disease** |  |  |  |
| Base SBP model | 12,076 | 0.653 (0.642 - 0.665) | Reference |
| Base model + SBP TITRE |  |  |  |
| Target: SBP<120mmHg | 12,051 | 0.657 (0.646 - 0.669) | 11.9% (7.7% - 16.1%) |
| Target: SBP<130mmHg | 12,026 | 0.659 (0.648 - 0.670) | 10.7% (6.5% - 14.8%) |
| Target: SBP<140mmHg | 12,010 | 0.660 (0.649 - 0.672) | 15.1% (11.5% - 20.0%) |
| Base model + SBP variability |  |  |  |
| SBP SD | 12,064 | 0.655 (0.644 - 0.667) | 0.6% (-3.6% - 4.9%) |
| SBP ARV | 12,061 | 0.655 (0.644 - 0.667) | 7.7% (3.4% - 11.9%) |
| Base DBP model | 12,290 | 0.624 (0.612 - 0.636) | Reference |
| Base model + DBP TITRE |  |  |  |
| Target: DBP<80mmHg | 12,259 | 0.628 (0.616 - 0.640) | 7.5% (3.3% - 11.7%) |
| Target: DBP<90mmHg | 12,247 | 0.630 (0.618 - 0.642) | 11.4% (7.3% - 15.5%) |
| Base model + DBP variability |  |  |  |
| DBP SD | 12,278 | 0.626 (0.614 - 0.638) | 5.8% (1.5% - 10.0%) |
| DBP ARV | 12,277 | 0.626 (0.614 - 0.638) | 9.1% (4.9% - 13.3%) |

*Base SBP model contained baseline SBP and other traditional risk factors, including age, sex, diabetes duration, HbA1c, body mass index, triglycerides, total cholesterol, high-density lipoprotein cholesterol, low-density lipoprotein cholesterol, history of cardiovascular disease, history of hypertension, and use of antihypertensive medications.

*Base DBP model contained baseline DBP and other traditional risk factors, including age, sex, diabetes duration, HbA1c, body mass index, triglycerides, total cholesterol, high-density lipoprotein cholesterol, low-density lipoprotein cholesterol, history of cardiovascular disease, history of hypertension, and use of antihypertensive medications.

In addition to the common covariates included in the models, baseline ba-PWV was additionally included in the model for arterial stiffness progression, and baseline eGFR was included in the model for incident DKD.

Abbreviations: SBP = systolic blood pressure, DBP = diastolic blood pressure.

| Table S14. Associations of cumulative BP load with the progression of arterial stiffness (n = 2,012) and DKD (n = 1,968) in patients with four fixed BP measurements | | | |
| --- | --- | --- | --- |
|  |  | **OR (95% CI)** | **P** |
| **Progression of Arterial Stiffness** |  |  |  |
| Cumulative SBP load |  |  |  |
| Target: SBP<120mmHg |  | 1.49 (1.30 - 1.72) | <0.001 |
| Target: SBP<130mmHg |  | 1.44 (1.25 - 1.67) | <0.001 |
| Target: SBP<140mmHg |  | 1.37 (1.18 - 1.61) | <0.001 |
| Cumulative DBP load |  |  |  |
| Target: DBP<80mmHg |  | 1.09 (0.97 - 1.23) | 0.162 |
| Target: DBP<90mmHg |  | 1.02 (0.91 - 1.13) | 0.772 |
| **Incident Diabetic Kidney Disease** |  |  |  |
| Cumulative SBP load |  |  |  |
| Target: SBP<120mmHg |  | 1.36 (1.18 - 1.56) | <0.001 |
| Target: SBP<130mmHg |  | 1.33 (1.17 - 1.50) | <0.001 |
| Target: SBP<140mmHg |  | 1.29 (1.16 - 1.44) | <0.001 |
| Cumulative DBP load |  |  |  |
| Target: DBP<80mmHg |  | 1.17 (1.04 - 1.33) | 0.011 |
| Target: DBP<90mmHg |  | 1.07 (0.95 - 1.19) | 0.225 |

BP measurements were recorded at baseline, 6 (±1 month), 12 (±1 month), and 18 months (±1 month). The odds ratios (OR) and 95% confidence intervals (CI) were adjusted for baseline covariates including age, sex, diabetes duration, blood pressure, HbA1c, BMI, triglycerides, total cholesterol, high-density lipoprotein cholesterol, low-density lipoprotein cholesterol, history of cardiovascular disease, history of hypertension, and use of antihypertensive medications. Analyses for arterial stiffness progression were additionally adjusted for baseline ba-PWV, while models for incident DKD were adjusted for baseline eGFR.

| Table S15. Prognostic value of cumulative BP load compared with traditional risk factors in patients with four fixed BP measurements | | | |
| --- | --- | --- | --- |
|  | **Akaike Information Criterion** | **C-Statistic  (95% CI)** | **Continuous Net Reclassification Improvement** |
| **Progression of Arterial Stiffness** |  |  |  |
| Base SBP model | 2,504 | 0.718 (0.696 - 0.740) | Reference |
| Base SBP model + cumulative SBP load |  |  |  |
| Target: SBP<120mmHg | 2,474 | 0.730 (0.708 - 0.752) | 24.2% (15.5% - 32.8%) |
| Target: SBP<130mmHg | 2,481 | 0.728 (0.706 - 0.750) | 21.0% (12.4% - 29.6%) |
| Target: SBP<140mmHg | 2,489 | 0.726 (0.704 - 0.748) | 8.7% (0.3% - 17.2%) |
| Base DBP model | 2,533 | 0.709 (0.686 - 0.731) | Reference |
| Base DBP model + cumulative DBP load |  |  |  |
| Target: DBP<80mmHg | 2,531 | 0.711 (0.688 - 0.733) | 18.7% (14.6% - 22.7%) |
| Target: DBP<90mmHg | 2,531 | 0.710 (0.687 - 0.732) | 13.2% (4.6% - 21.8%) |
| **Incident Diabetic Kidney Disease** |  |  |  |
| Base SBP model | 2,137 | 0.644 (0.616 - 0.672) | Reference |
| Base SBP model + cumulative SBP load |  |  |  |
| Target: SBP<120mmHg | 2,120 | 0.656 (0.627 - 0.684) | 16.4% (6.2% - 26.6%) |
| Target: SBP<130mmHg | 2,119 | 0.655 (0.626 - 0.683) | 14.1% (9.5% - 22.3%) |
| Target: SBP<140mmHg | 2,118 | 0.653 (0.625 - 0.682) | 10.1% (4.4% - 17.5%) |
| Base DBP model | 2,139 | 0.640 (0.612 - 0.668) | Reference |
| Base DBP model + cumulative DBP load |  |  |  |
| Target: DBP<80mmHg | 2,135 | 0.644 (0.616 - 0.672) | 13.9% (6.8% - 19.7%) |
| Target: DBP<90mmHg | 2,139 | 0.641 (0.612 - 0.669) | 12.8% (3.0% - 22.7%) |

BP measurements were recorded at baseline, 6 (±1 month), 12 (±1 month), and 18 months (±1 month).

*Base SBP model contained baseline SBP and other traditional risk factors, including age, sex, diabetes duration, HbA1c, body mass index, triglycerides, total cholesterol, high-density lipoprotein cholesterol, low-density lipoprotein cholesterol, and history of cardiovascular disease.

*Base DBP model contained baseline DBP and other traditional risk factors, including age, sex, diabetes duration, HbA1c, body mass index, triglycerides, total cholesterol, high-density lipoprotein cholesterol, low-density lipoprotein cholesterol, and history of cardiovascular disease.

In addition to the common covariates included in the models, baseline ba-PWV was additionally included in the model for arterial stiffness progression, and baseline eGFR was included in the model for incident DKD.

Abbreviations: SBP = systolic blood pressure, DBP = diastolic blood pressure.

| Table S16. Associations of cumulative BP load with the progression of arterial stiffness (n = 2,243) and DKD (n = 2,037) in patients whose BP was measured using the OMRON HBP-9031C device | | | |
| --- | --- | --- | --- |
|  |  | **OR (95% CI)** | **P** |
| **Progression of Arterial Stiffness** |  |  |  |
| Cumulative SBP load |  |  |  |
| Target: SBP<120mmHg |  | 1.63 (1.41 - 1.88) | <0.001 |
| Target: SBP<130mmHg |  | 1.52 (1.30 - 1.79) | <0.001 |
| Target: SBP<140mmHg |  | 1.43 (1.20 - 1.72) | <0.001 |
| Cumulative DBP load |  |  |  |
| Target: DBP<80mmHg |  | 1.32 (1.18 - 1.47) | <0.001 |
| Target: DBP<90mmHg |  | 1.12 (1.02 - 1.24) | 0.002 |
| **Incident Diabetic Kidney Disease** |  |  |  |
| Cumulative SBP load |  |  |  |
| Target: SBP<120mmHg |  | 1.24 (1.08 - 1.43) | 0.003 |
| Target: SBP<130mmHg |  | 1.16 (1.01 - 1.34) | 0.034 |
| Target: SBP<140mmHg |  | 1.09 (0.95 - 1.25) | 0.226 |
| Cumulative DBP load |  |  |  |
| Target: DBP<80mmHg |  | 1.12 (0.98 - 1.26) | 0.081 |
| Target: DBP<90mmHg |  | 1.06 (0.94 - 1.17) | 0.311 |

The odds ratios (OR) and 95% confidence intervals (CI) were adjusted for baseline covariates including age, sex, diabetes duration, blood pressure, HbA1c, BMI, triglycerides, total cholesterol, high-density lipoprotein cholesterol, low-density lipoprotein cholesterol, history of cardiovascular disease, history of hypertension, and use of antihypertensive medications. In addition, the number of BP measurements was included. Analyses for arterial stiffness progression were additionally adjusted for baseline ba-PWV, while models for incident DKD were adjusted for baseline eGFR.

| Table S17. Prognostic value of cumulative BP load compared with traditional risk factors in patients whose BP was measured using the OMRON HBP-9031C device | | | |
| --- | --- | --- | --- |
|  | **Akaike Information Criterion** | **C-Statistic  (95% CI)** | **Continuous Net Reclassification Improvement** |
| **Progression of Arterial Stiffness** |  |  |  |
| Base SBP model | 2,948 | 0.715 (0.695 - 0.736) | Reference |
| Base SBP model + cumulative SBP load |  |  |  |
| Target: SBP<120mmHg | 2,902 | 0.730 (0.709 - 0.750) | 29.7% (21.8% - 37.7%) |
| Target: SBP<130mmHg | 2,921 | 0.725 (0.704 - 0.745) | 16.4% (12.4% - 24.3%) |
| Target: SBP<140mmHg | 2,933 | 0.721 (0.701 - 0.742) | 6.5% (0.2% - 13.2%) |
| Base DBP model | 2,948 | 0.716 (0.696 - 0.737) | Reference |
| Base DBP model + cumulative DBP load |  |  |  |
| Target: DBP<80mmHg | 2,923 | 0.724 (0.703 - 0.744) | 22.7% (14.9% - 30.5%) |
| Target: DBP<90mmHg | 2,943 | 0.718 (0.698 - 0.739) | 20.7% (12.9% - 28.4%) |
| **Incident Diabetic Kidney Disease** |  |  |  |
| Base SBP model | 1,980 | 0.658 (0.627 - 0.688) | Reference |
| Base SBP model + cumulative SBP load |  |  |  |
| Target: SBP<120mmHg | 1,972 | 0.665 (0.635 - 0.695) | 15.9% (5.2% - 26.6%) |
| Target: SBP<130mmHg | 1,977 | 0.661 (0.631 - 0.692) | 13.3% (7.6% - 19.6%) |
| Target: SBP<140mmHg | 1,979 | 0.659 (0.628 - 0.689) | 7.2% (1.3% - 14.0%) |
| Base DBP model | 1,986 | 0.653 (0.622 - 0.684) | Reference |
| Base DBP model + cumulative DBP load |  |  |  |
| Target: DBP<80mmHg | 1,984 | 0.656 (0.625 - 0.686) | 13.6% (3.0% - 24.1%) |
| Target: DBP<90mmHg | 1,985 | 0.654 (0.623 - 0.685) | 6.7% (0.4% - 13.7%) |

*Base SBP model contained baseline SBP and other traditional risk factors, including age, sex, diabetes duration, HbA1c, body mass index, triglycerides, total cholesterol, high-density lipoprotein cholesterol, low-density lipoprotein cholesterol, and history of cardiovascular disease.

*Base DBP model contained baseline DBP and other traditional risk factors, including age, sex, diabetes duration, HbA1c, body mass index, triglycerides, total cholesterol, high-density lipoprotein cholesterol, low-density lipoprotein cholesterol, and history of cardiovascular disease.

In addition to the common covariates included in the models, baseline ba-PWV was additionally included in the model for arterial stiffness progression, and baseline eGFR was included in the model for incident DKD.

Abbreviations: SBP = systolic blood pressure, DBP = diastolic blood pressure.

Table S18. STROBE Statement—Checklist of items that should be included in reports of cohort studies

|  | Item No | Recommendation | Page No |
| --- | --- | --- | --- |
| **Title and abstract** | 1 | (*a*) Indicate the study’s design with a commonly used term in the title or the abstract | 1, 3-4 |
|  |  | (*b*) Provide in the abstract an informative and balanced summary of what was done and what was found |  |
| Introduction | | | |
| Background/rationale | 2 | Explain the scientific background and rationale for the investigation being reported | 5-6 |
| Objectives | 3 | State specific objectives, including any prespecified hypotheses | 6 |
| Methods | | | |
| Study design | 4 | Present key elements of study design early in the paper | 7 |
| Setting | 5 | Describe the setting, locations, and relevant dates, including periods of recruitment, exposure, follow-up, and data collection | 7 |
| Participants | 6 | (*a*) Give the eligibility criteria, and the sources and methods of selection of participants. Describe methods of follow-up |  |
|  |  | (*b*) For matched studies, give matching criteria and number of exposed and unexposed | 7 |
| Variables | 7 | Clearly define all outcomes, exposures, predictors, potential confounders, and effect modifiers. Give diagnostic criteria, if applicable | 8-9 |
| Data sources/ measurement | 8* | For each variable of interest, give sources of data and details of methods of assessment (measurement). Describe comparability of assessment methods if there is more than one group | 8-9  Supplementary Methods |
| Bias | 9 | Describe any efforts to address potential sources of bias | 10-14 |
| Study size | 10 | Explain how the study size was arrived at | 13 |
| Quantitative variables | 11 | Explain how quantitative variables were handled in the analyses. If applicable, describe which groupings were chosen and why | 10 |
| Statistical methods | 12 | (*a*) Describe all statistical methods, including those used to control for confounding |  |
|  |  | (*b*) Describe any methods used to examine subgroups and interactions | 10-13 |
|  |  | (*c*) Explain how missing data were addressed |  |
|  |  | (*d*) If applicable, explain how loss to follow-up was addressed |  |
|  |  | (*e*) Describe any sensitivity analyses |  |
| Results | | |  |
| Participants | 13* | (a) Report numbers of individuals at each stage of study—eg numbers potentially eligible, examined for eligibility, confirmed eligible, included in the study, completing follow-up, and analysed | 14 |
|  |  | (b) Give reasons for non-participation at each stage |  |
|  |  | (c) Consider use of a flow diagram |  |
| Descriptive data | 14* | (a) Give characteristics of study participants (eg demographic, clinical, social) and information on exposures and potential confounders | 14 |
|  |  | (b) Indicate number of participants with missing data for each variable of interest |  |
|  |  | (c) Summarise follow-up time (eg, average and total amount) |  |
| Outcome data | 15* | Report numbers of outcome events or summary measures over time | 14 |

| Main results | 16 | (*a*) Give unadjusted estimates and, if applicable, confounder-adjusted estimates and their precision (eg, 95% confidence interval). Make clear which confounders were adjusted for and why they were included | 14-18 |
| --- | --- | --- | --- |
|  |  | (*b*) Report category boundaries when continuous variables were categorized |  |
|  |  | (*c*) If relevant, consider translating estimates of relative risk into absolute risk for a meaningful time period |  |
| Other analyses | 17 | Report other analyses done—eg analyses of subgroups and interactions, and sensitivity analyses | 18-19 |
| Discussion | | | |
| Key results | 18 | Summarise key results with reference to study objectives | 19 |
| Limitations | 19 | Discuss limitations of the study, taking into account sources of potential bias or imprecision. Discuss both direction and magnitude of any potential bias | 23 |
| Interpretation | 20 | Give a cautious overall interpretation of results considering objectives, limitations, multiplicity of analyses, results from similar studies, and other relevant evidence | 22 |
| Generalisability | 21 | Discuss the generalisability (external validity) of the study results | 24 |
| Other information | | | |
| Funding | 22 | Give the source of funding and the role of the funders for the present study and, if applicable, for the original study on which the present article is based | 26 |

*Give information separately for exposed and unexposed groups.

**Note:** An Explanation and Elaboration article discusses each checklist item and gives methodological background and published examples of transparent reporting. The STROBE checklist is best used in conjunction with this article (freely available on the Web sites of PLoS Medicine at http://www.plosmedicine.org/, Annals of Internal Medicine at http://www.annals.org/, and Epidemiology at http://www.epidem.com/). Information on the STROBE Initiative is available at http://www.strobe-statement.org.

Reference

1. Bi Y, Jiang Y, He J, Xu Y, Wang L, Xu M, Zhang M, Li Y, Wang T, Dai M *et al*: **Status of cardiovascular health in Chinese adults**. *J Am Coll Cardiol* 2015, **65**(10):1013-1025.

2. Colberg SR, Sigal RJ, Yardley JE, Riddell MC, Dunstan DW, Dempsey PC, Horton ES, Castorino K, Tate DF: **Physical Activity/Exercise and Diabetes: A Position Statement of the American Diabetes Association**. *Diabetes Care* 2016, **39**(11):2065-2079.
